# B cell receptor signaling in germinal centers prolongs survival and primes B cells for selection

**DOI:** 10.1016/j.immuni.2023.02.003

**Published:** 2023-03-06

**Authors:** Spencer T. Chen, Thiago Y. Oliveira, Anna Gazumyan, Melissa Cipolla, Michel C. Nussenzweig

**Affiliations:** 1 Laboratory of Molecular Immunology, The Rockefeller University; New York, NY 10065, USA.; 2 Howard Hughes Medical Institute.; 3 Lead Contact.

## Abstract

Germinal centers (GCs) are sites of B cell clonal expansion, diversification, and antibody affinity selection. This process is limited and directed by T follicular helper cells that provide helper signals to B cells that endocytose, process, and present cognate antigens in proportion to their B cell receptor (BCR) affinity. Under this model, the BCR functions as an endocytic receptor for antigen capture. How signaling through the BCR contributes to selection is not well understood. To investigate the role of BCR signaling in GC selection, we developed a tracker for antigen binding and presentation and a Bruton’s tyrosine kinase drug-resistant-mutant mouse model. We showed that BCR signaling per se is necessary for the survival and priming of light zone B cells to receive T cell help. Our findings provide insight into how high-affinity antibodies are selected within GCs and are fundamental to our understanding of adaptive immunity and vaccine development.

## Graphical Abstract

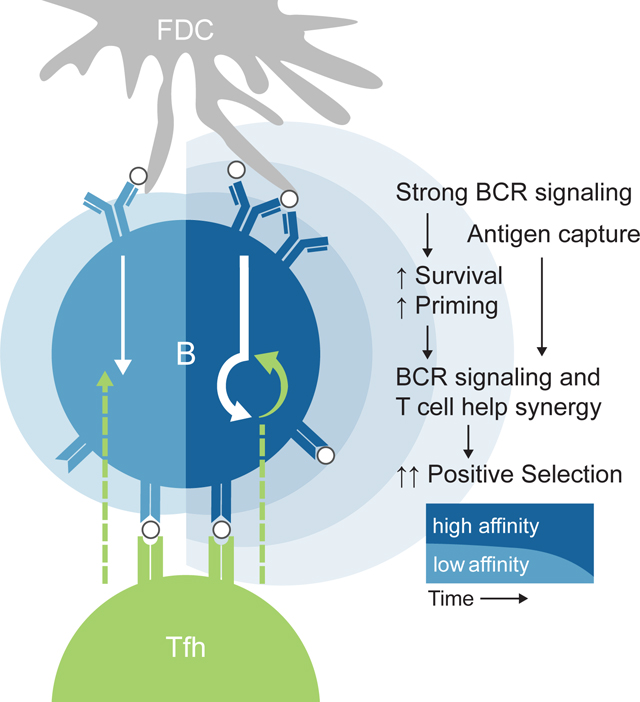

The role that B cell receptor (BCR) signaling plays in selection within germinal centers (GCs) is not well understood. Here, Chen et al. demonstrate that BCR signaling is necessary for the survival and priming of light zone (LZ) B cells and that these signals synergize with T cell help to enhance positive selection of GC B cells.

During adaptive immune responses, B cells undergo clonal expansion, antibody gene diversification, and affinity selection in GCs. Within the GC, B cells differentiate into protective antibody-producing plasma and memory B cells essential for long-lived immunity^1,2^. Understanding how these events are controlled and how high-affinity clones are selected within the GC is fundamental to our understanding of adaptive immunity and of crucial importance to the development of vaccines.

GCs are divided into two zones: a light zone (LZ); and a dark zone (DZ)^3^. A working model for affinity-based selection stipulates that antigen displayed on follicular dendritic cells (FDCs) in the LZ^4,5^ is captured by BCRs, internalized, processed, and presented to Tfh cells that select B cells that display higher levels of cognate peptides on major histocompatibility molecules (pMHC)^6^. According to this model, GC selection is determined primarily by the ability of the BCR to bind to and endocytose antigen. However, the BCR is a dual-purpose receptor that is both a signal transducer and an endocytic receptor, and the role of BCR signaling in affinity-based selection remains to be precisely understood.

Experiments with isolated GC B cells initially indicated that they are largely insensitive to soluble antigen^7^. This finding, along with the lower levels of surface BCR expression on GC B cells^2^, led to the view that GC BCR signaling is silenced *in vivo*. However, more recent work, with a Nur77-eGFP reporter and experiments in which GC B cells were exposed to membrane-tethered antigen that resembles the display on FDCs, showed that GC B cells signal through the BCR *in vivo* and *ex vivo*^8–10^, albeit through altered signaling pathways compared to naive B cells^7,9,11,12^. The implications of these altered signaling pathways and whether BCR signaling per se plays a direct role in selection remains to be determined.

Here we report on the development of a molecular tracker of *in vivo* antigen binding and presentation and a novel drug-resistant mouse model that we used to examine the role of BCR signaling in GC selection. The data indicate that continuous BCR signaling primes LZ B cells to receive positive selection signals from Tfh and is also necessary for their survival. Therefore, both BCR signaling and endocytosis are required for the selection of high-affinity cells vital to antibody-mediated immune protection during natural infection and vaccination.

## Results:

### NP-Eα tracking identifies GC B cells engaging antigen *in vivo.*

**Figure 1.**
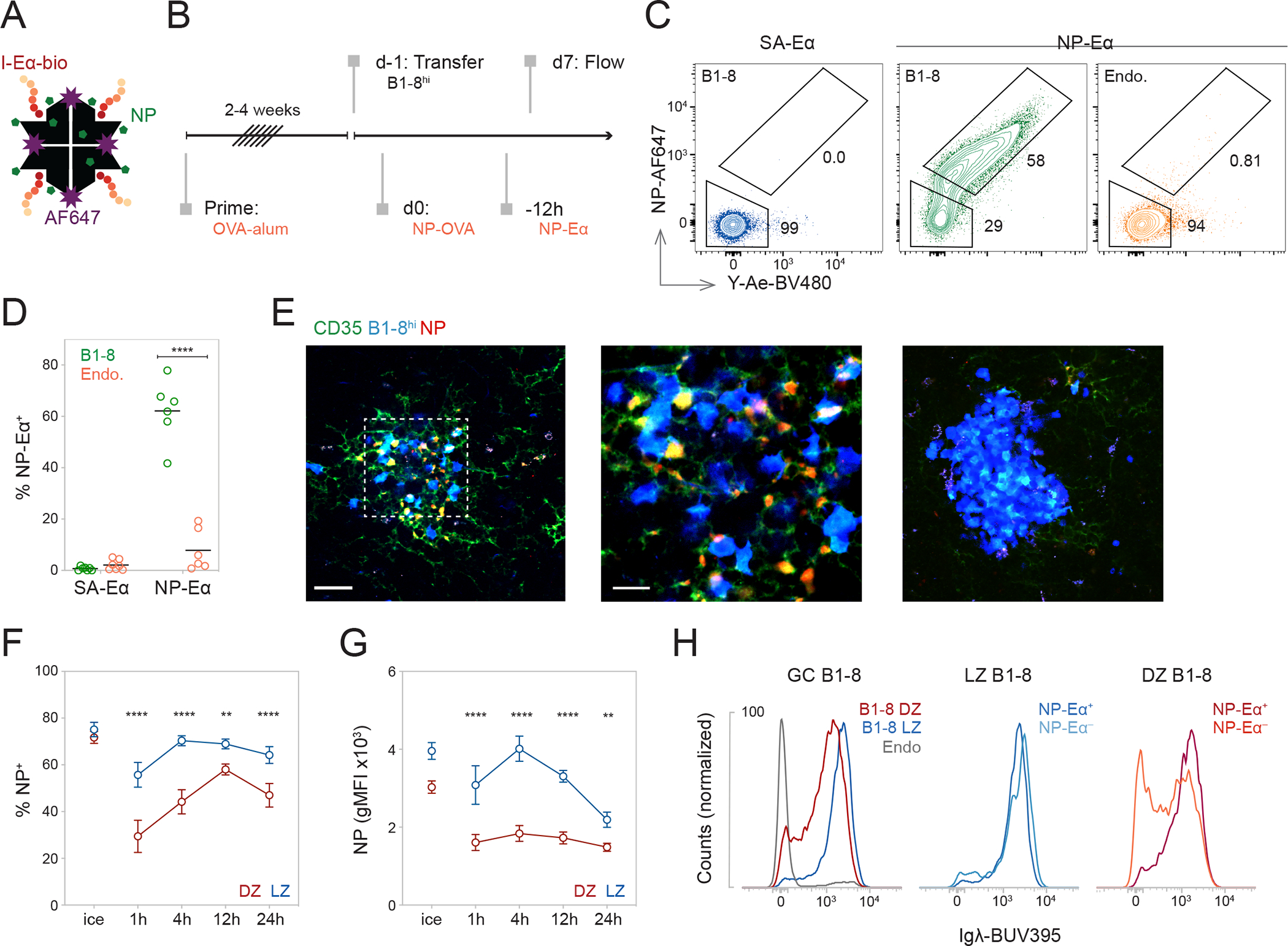
NP-Eα tracks antigen binding and presenting GC B cells in a BCR-specific manner. **(A)** Cartoon representation of NP-Eα. **(B)** Experimental setup. **(C)** Representative flow cytometry plots showing internalization and presentation of SA-Eα or NP-Eα by GC B cell populations. **(D)** Frequency of NP^+^Y-Ae^+^ (NP-Eα^+^) among B1-8^hi^ or host (Endo.) B cells after injection of ^7^NP-Eα or SA-Eα, ****p<0.0001. **(E)** Multiphoton images of GCs after prime-boost and transfer of B1-8^hi^-CFP cells. αCD35-AF488 and ^7^NP-Eα-AF594 were injected intravenously (i.v.) and subcutaneously (s.c.), respectively, 24 hours (h) before imaging. LZs were identified by the presence of FDC networks labeled with αCD35. LZ (leftmost panel); inset of LZ as marked with the dashed line (center); and DZ (rightmost panel). **(F)** Percentage of NP^+^ cells (**p=0.0010, ****p<0.0001) and **(G)** Geometric mean fluorescence intensity (gMFI) of NP-AF647 (**p=0.0023, ****p<0.0001) of DZ and LZ labeled with ^7^NP-Eα on ice or *in vivo* over time **(H)** Representative histograms showing Igλ expression of GC populations. (D, F, and G) Data from two independent experiments. Each dot represents one mouse and lines depict mean (D). Dots represent means and error bars, SEM (F and G). P values calculated using two-tailed paired t test (D) and RM two-way ANOVA with Šidák’s multiple comparisons (F and G). See also Figure S1.

To track antigen binding and processing by GC B cells *in vivo*, we produced a tetrameric antigen consisting of fluorescently labeled streptavidin (SA-AF647) coupled to 4-hydroxy-3-nitrophenylacetyl (NP) and biotinylated I-E_52–73_ (Eα) peptide (NP-Eα) (Figure 1A). NP-specific B cells that bind and internalize NP-Eα will be AF647 fluorescent, and those that process and present the antigen as pMHC can be detected with an antibody specific to the Eα-pMHC (Y-Ae)^13–15^.

To test this approach, we elicited GC reactions using congenically-marked B cells carrying a knock-in heavy chain that, when paired with a lambda light chain (Igλ), produces a high-affinity receptor for NP (B1-8^hi^)^16^. B1-8^hi^ B cells were adoptively transferred into ovalbumin (OVA)-primed mice that were subsequently boosted with NP-conjugated OVA (NP-OVA) (Figures 1B and S1A). This immunization scheme produces GCs containing OVA-specific Tfh cells, NP-specific B1-8^hi^ B cells, and host B cells^17^. The small amount of low-valency NP-Eα used for *in vivo* tracking produced no measurable increase in apoptosis (Figure S1B). Under these conditions, 40–80% of B1-8^hi^ GC cells were AF647 labeled, and cells that bound NP-Eα also presented it as indicated by staining with Y-Ae (NP-Eα^+^) (Figures 1C and 1D). Control SA-AF647 labeled tetramers without NP showed little or no direct fluorescence staining (Figures 1C and 1D). Imaging GCs revealed that NP-Eα is localized to FDCs in the LZ and bound and internalized by B1-8^hi^ cells (Figure 1E). We conclude that the NP-Eα tracker identifies B cells binding and presenting antigen *in vivo*.

Notably, we failed to detect NP-Eα binding and presentation by 15–40% of B1-8^hi^ cells in GCs. To investigate the kinetics of NP-Eα tracking *in vivo*, we introduced it into GC reactions at consecutive time points (Figures 1F, 1G, S1C–S1I). The relative proportion of B1-8^hi^ cells that failed to bind NP-Eα was consistently higher in the DZ than in the LZ (Figures 1F, S1D and S1F). Consistent with phenotypic and BCR surface expression differences between LZ and DZ cells^2^, the amount of antigen bound by LZ cells was higher (Figures 1G and S1G). To determine if the apparent lack of binding was a consequence of downregulated BCR expression, we measured surface BCR by staining for Igλ. Although, Igλ surface expression was comparable in LZ-NP-Eα^+^ and −NP-Eα^−^ cells, DZ-NP-Eα^−^ cells showed a bimodal distribution of surface BCR, which may reflect the accumulation of mutations resulting in nonfunctional BCRs and dilution of surface BCRs during DZ cell division (Figures 1H, S1E, S1H, and S1I)^18,19^. Consequently, the absence of antigen binding by some DZ but not LZ cells might be explained by lower surface BCR expression.

### Loss of antigen engagement *in vivo* is associated with deleterious somatic hypermutation.

**Figure 2.**
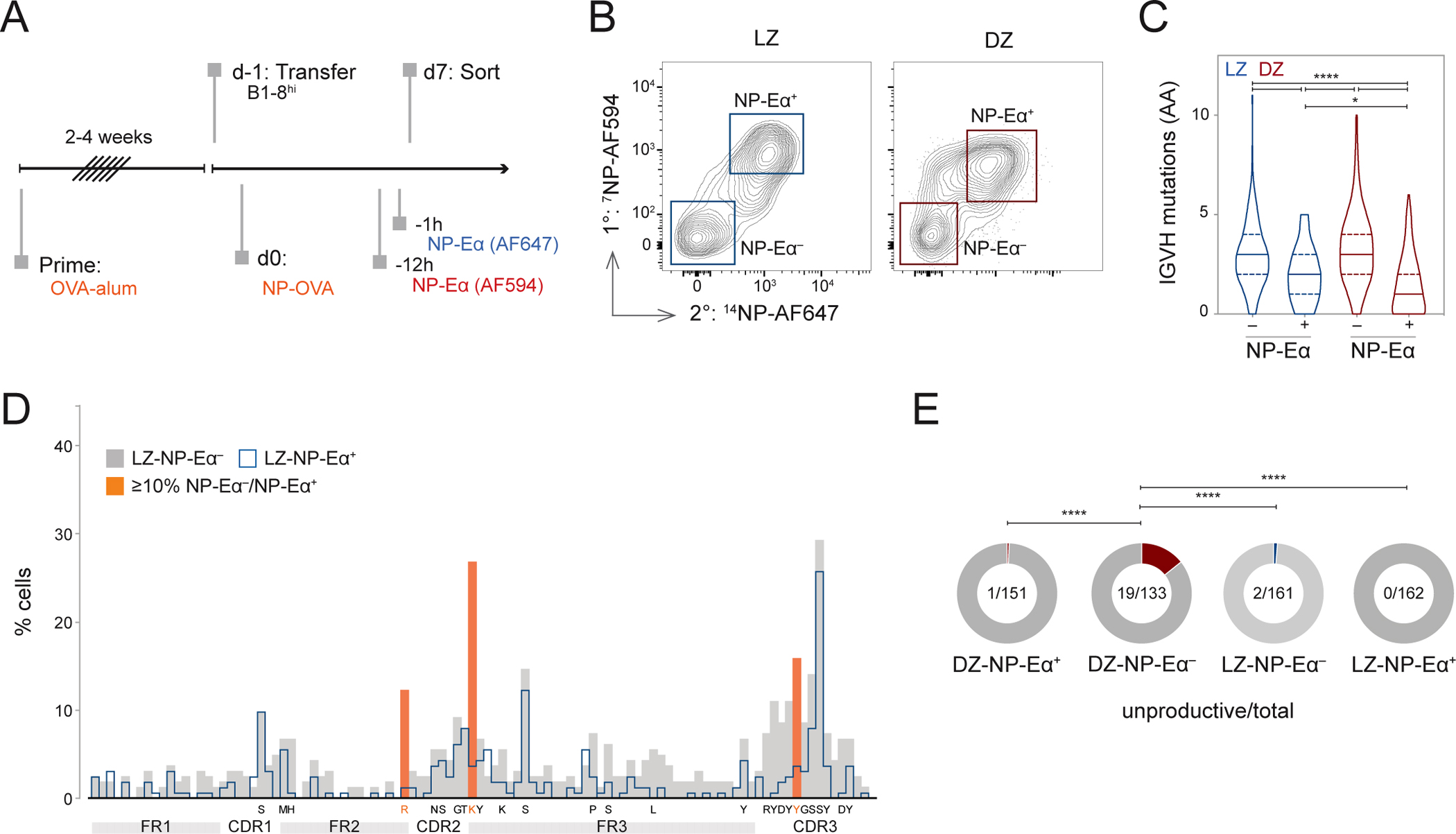
Mutation analysis of Fabs cloned from DZ and LZ compartments. **(A)** Experimental setup. ^7^NP-Eα-AF594 and ^14^NP-Eα-AF647 were injected 12h and 1h, respectively, before sacrifice. **(B)** Representative gating of sorted B1-8^hi^ LZ and DZ populations. **(C)** Number of amino acid (AA) mutations in IGVH chains of sorted populations, Violin plot depicts median and quartiles. *p=0.020, ****p<0.0001, Kruskall-Wallis with Dunn’s multiple corrections test. **(D)** Distribution of mutations in LZ-NP-Eα^−^ and LZ-NP-Eα^+^ populations. AAs targeted in 5% or more of LZ-NP-Eα^−^ over LZ-NP-Eα^+^ populations listed below axis; those targeted 10% or more are highlighted in orange. **(E)** Fraction of unproductive IGVH chains by compartment, Fisher’s exact test, ****p<0.0001. See also Figure S2.

To determine why a fraction of GC B1-8^hi^-Igλ^+^ B cells do not detectably bind NP-Eα we purified them following sequential injections of NP-Eα—to maximize tracking—and sequenced their *Ig* genes (Figure 2A). Two groups of LZ and DZ cells were examined: double negative cells (LZ-NP-Eα^−^ and DZ-NP-Eα^−^) that were not labeled; and double positive cells (LZ-NP-Eα^+^ and DZ-NP-Eα^+^) that were (Figures 2B). NP-Eα^−^ cells were more mutated than their antigen-binding counterparts (Figures 2C, 2D, and S2A) and were also less likely to express the “germline” knock-in IGVH gene (Figure S2B). Consistent with a DZ selection checkpoint for BCR expression^18,19^, non-productive *Ig* sequences containing stop or frameshift mutations were significantly enriched in the DZ-NP-Eα^−^ compartment and rarely found in LZ cells (p<0.0001) (Figure 2E). LZ-NP-Eα^−^ cells also showed lower frequencies of mutations in FR3 and CDR3 when compared to DZ-NP-Eα^−^ cells (Figure S2C). Analysis of the mutational landscape of LZ cells revealed an accumulation of R55G and K66E, K66N, or K66Q replacements in LZ-NP-Eα^−^ cells, implicating these replacements in the loss of binding (Figures 2D and S2D). We conclude that loss of measurable antigen binding by flow cytometry is associated with deleterious somatic hypermutation (SHM).

**Figure 3.**
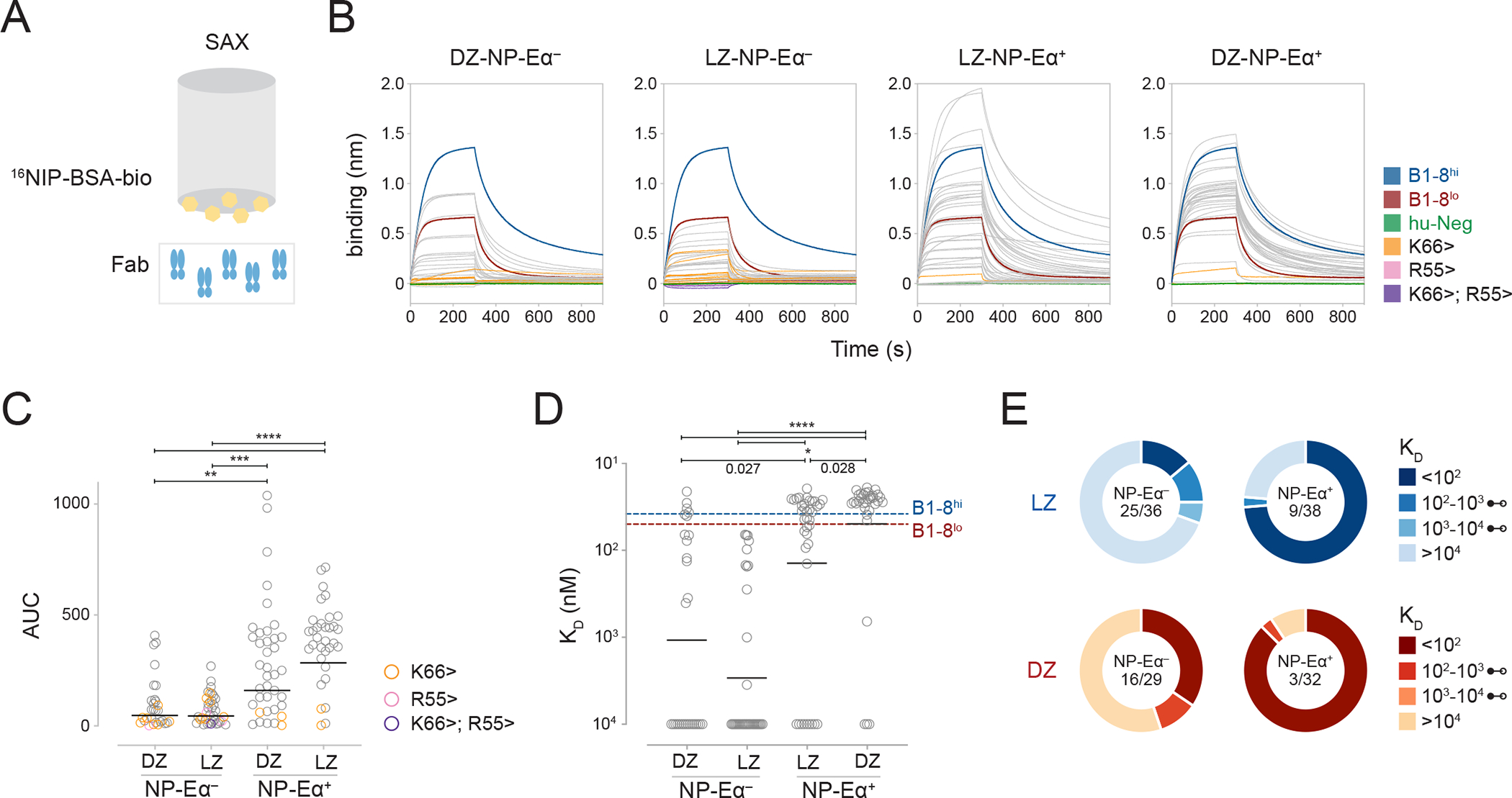
Binding affinities of Fabs produced from LZ and DZ B cells. **(A)** Monovalent bio-layer interferometry (BLI) setup. **(B)** BLI traces of Fabs [50nM] from LZ- and DZ-NP-Eα^+^ and -NP-Eα^−^ compartments under monovalent setup. **(C)** Area Under the Curve (AUC) calculations of BLI traces from (B), **p=0.0027, ***p=0.0002, ****p<0.0001. **(D)** K_D_ measurements of Fabs from LZ- and DZ-NP-Eα^+^ and ^−^NP-Eα^−^ compartments. Fabs with no detectable binding were assigned K_D_ values of 10 μM, *p values as indicated, ****p<0.0001. **(E)** Distribution of K_D_ values of Fabs from LZ and DZ NP-Eα^+^ and NP-Eα^−^ compartments. Fraction in middle denotes the number of Fabs with undetectable binding out of the total. Each dot represents one Fab with lines denoting geometric means (C and D). P values (C and D) calculated with Kruskall-Wallis with Dunn’s multiple corrections test. See also Figure S2.

To determine whether mutations associated with absence of NP-Eα binding impact affinity, we cloned and produced antibodies expressed by LZ and DZ cells and performed bio-layer interferometry (BLI) (Figures 3A, 3B, and S2E–S2K). Monovalent interactions were modeled by coupling ^16^NIP-BSA-biotin to the sensor and using Fabs as the analyte (Figures 3B–3E). Control B1-8^hi^ and its lower-affinity variant, B1-8^lo^, Fabs showed K_D_s of 38nM and 50nM, respectively in this assay (Figure 3D)^15,16^. Fabs obtained from LZ- and DZ-NP-Eα^+^ cells showed relatively high affinities with geometric mean K_D_ values of 141nM and 49nM, respectively (Figure 3D). Among the 29 Fabs from DZ-NP-Eα^−^ cells, 10 showed affinities in the range of B1-8^hi^, suggesting that some DZ-NP-Eα^−^ cells that fail to bind NP-Eα *in vivo* encode BCRs with binding capacities (Figures 1H, 2B, 3D, and 3E). In contrast, all 36 Fabs from LZ-NP-Eα^−^ cells showed lower affinities than B1-8^lo^ with a geometric mean K_D_ value of 2.9μM (Figures 3D and 3E). Accumulation of IGVH mutations was negatively correlated with affinity (Figure S2F) and antibodies with mutations in either R55 or K66, which are enriched among LZ nonbinders (Figure S2D), showed no measurable binding (Figures 3B and 3C). To model multivalent interactions found *in vivo*, we immobilized Fabs onto sensors and measured binding to multivalent antigen (Figures S2G–S2K). Of the 25 Fabs derived from LZ-NP-Eα^−^ cells with undetectable monovalent binding, 18 bound to the higher valency substrate, but only one reached the apparent binding affinity of B1-8^lo^ (Figure S2J). Thus, flow cytometry with NP-Eα fails to capture low-affinity interactions that are detectable by multimerized antigen in BLI assays. Nevertheless, NP-Eα engagement is an indicator of the relative antigen binding affinity of LZ cells.

### Positive selection is enhanced among cells with active BCR engagement.

**Figure 4.**
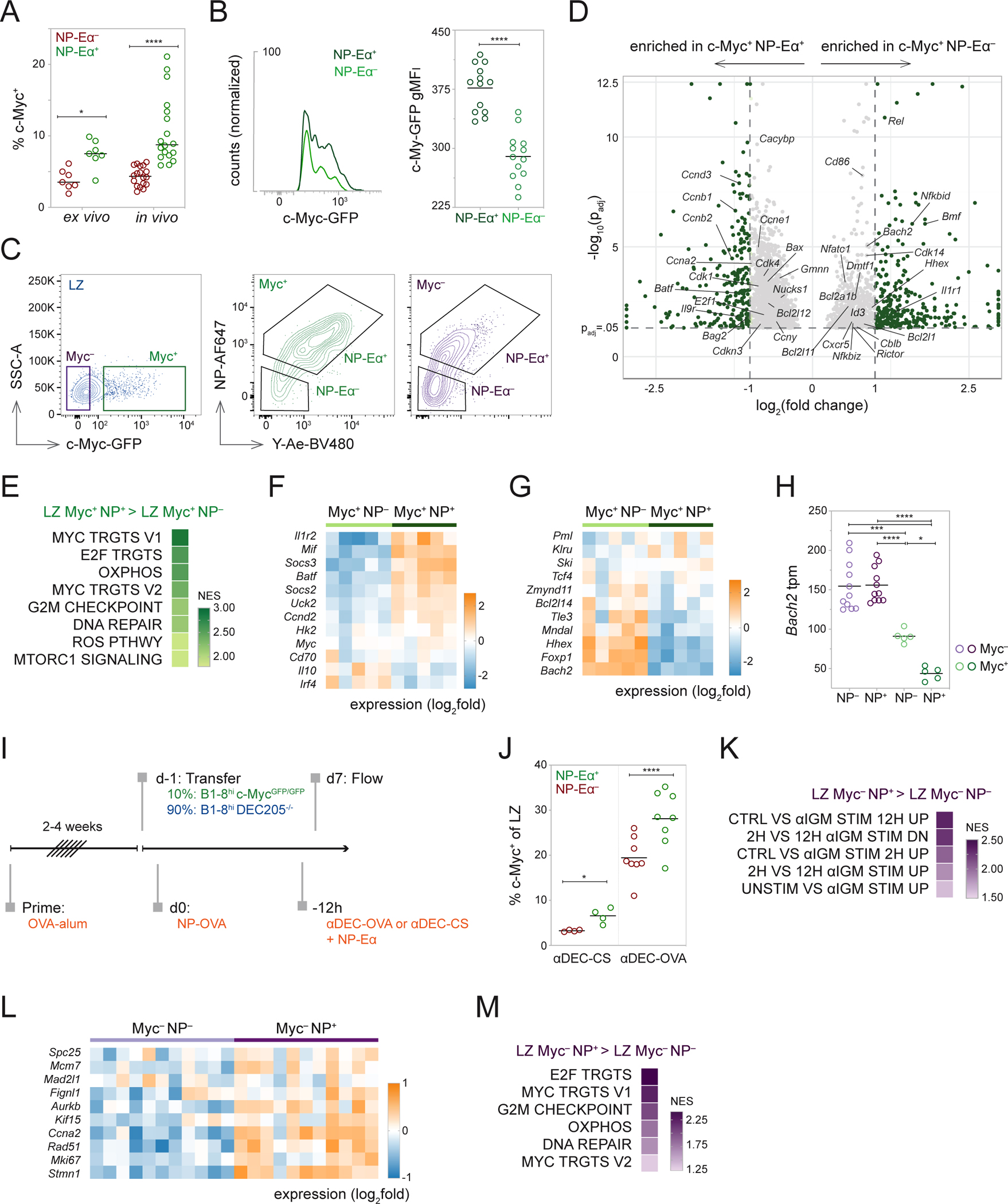
Transcriptome analysis of pathways induced upon BCR engagement and positive selection. **(A)** Frequency of c-Myc^+^ cells among NP-Eα^+^ and NP-Eα^−^ LZ B1-8^hi^ cells stained *ex vivo* on ice or *in vivo*, two-way ANOVA with Šidák’s multiple comparisons, *p=0.0411, ****p<0.0001. **(B)** Representative histograms showing c-Myc-GFP expression in NP-Eα binding and nonbinding LZ B1-8^hi^ cells (left) and summary of gMFI intensities (right), ****p<0.0001. **(C)** Sorting strategy for c-Myc^+^ NP-Eα^+^, c-Myc^+^ NP-Eα^−^, c-Myc^−^ NP-Eα^+^, and c-Myc^−^ NP-Eα^−^ populations. **(D)** Volcano plot depicting snapshot of differentially expressed genes between c-Myc^+^ populations, genes with p_adj_>0.05 not shown. Genes with log_2_(fold change) >3 and <−3 plotted as log_2_(3) and log_2_(−3), respectively. Genes with −log_10_(p_adj_)>12.5 plotted as −log_10_(12.5). **(E)** Gene Set Enrichment Analysis (GSEA) summary of enriched hallmark pathways. **(F)** Heatmap depicting expression of “immune activation” genes, and **(G)** transcription factors associated with memory B cell differentiation among c-Myc^+^ NP-Eα^+^ and c-Myc^+^ NP-Eα^−^ populations. **(H)** Expression of *Bach2* mRNA, one-way ANOVA with Tukey’s multiple comparisons test, *p=0.0161, ***p=0.0001, ****p<0.0001. **(I)** Experimental setup for αDEC205 targeting. **(J)** Frequency of c-Myc^+^ cells among LZ NP-Eα^+^ and NP-Eα^−^ cells targeted with αDEC-CS (negative control, left) or αDEC-OVA (right), *p=0.0318, ****p<0.0001. **(K)** GSEA summary of BCR stimulation pathways, **(L)** Heatmap depicting expression of BCR stimulation genes, and **(M)** GSEA summary of enriched hallmark pathways between c-Myc^−^ NP-Eα^+^ and c-Myc^−^ NP-Eα^−^ populations. Data from two (J), four (A), and five (B) independent experiments. Each dot represents one mouse (A, B, and J). Each dot (H) or square (F, G, and L) represents a population of 400 cells. (A, B, H, and J) Lines depict means. P values (B and J) calculated with two-tailed paired t test. (E, K, and M) All enriched pathways had nominal p values < 0.05 and FDR q values < 0.25. See also Figures S3 and S4.

*Myc* expression marks LZ cells that received Tfh activation signals associated with positive selection^20,21^. To examine the role of BCR signaling in LZ B cell selection, we used a c-Myc-green fluorescent protein (GFP) reporter (B1-8^hi^ c-Myc-GFP)^20,22^ and tracked antigen binding by injection of NP-Eα (Figure S3A). B1-8^hi^ tracking by NP-Eα confers no additional T cell selection advantage because processing and presentation of NP-Eα provides no cognate antigen for presentation to OVA-specific Tfh. As expected, the fraction of c-Myc^+^ cells was significantly higher among LZ-NP-Eα^+^ that retain the ability to bind NP when compared to LZ-NP-Eα^−^ cells, irrespective of whether NP-Eα staining was done *in vivo* or *ex vivo* (Figures 4A and S3B). Furthermore, *Myc* expression by LZ-NP-Eα^+^ cells was higher as measured by their GFP mean fluorescence intensity (MFI) (Figure 4B). Similarly, the amount of antigen captured, as measured by MFI, was higher among LZ c-Myc^+^ NP-Eα^+^ cells when compared to LZ c-Myc^−^ NP-Eα^+^ cells (Figure S3C). Therefore, *Myc* expression and, by inference, positive selection, are enriched among LZ cells that bind antigen with higher affinity.

To contextualize the molecular pathways induced upon BCR engagement in the GC, we isolated four populations of LZ B cells based on their relative affinity for antigen and c-Myc expression and performed bulk mRNA-seq: c-Myc^−^ NP-Eα^+^; c-Myc^−^ NP-Eα^−^; c-Myc^+^ NP-Eα^+^; and c-Myc^+^ NP-Eα^−^ (Figures 4C, S3D, and S3E).

We initially compared the transcriptomes of c-Myc^+^ LZ cells that did or did not detectably bind antigen (Figure 4D). Gene Set Enrichment Analysis (GSEA) showed that c-Myc^+^ NP-Eα^+^ cells were enriched in pathways induced by c-Myc, mTOR, and Nuclear Factor-κB (NF-κB) relative to lower affinity c-Myc^+^ NP-Eα^−^ cells (Figure S3F). c-Myc^+^ NP-Eα^+^ cells also showed enriched expression of hallmark pathways associated with cell-cycle entry and energy metabolism (Figures 4E and S3G)^23^. In addition to cell-cycle entry and control genes like *Ccnd2* and *Batf*, higher affinity cells showed greater expression of immune activation genes involved in cytokine responses such as *Il1r2*, *Socs2*, and *Socs3*, and genes involved in metabolic regulation, *Uck2* (Figures 4F and S3H)^20^. Altogether, the transcriptional profile of the c-Myc^+^ NP-Eα^+^ population suggests these cells have received stronger selection signals relative to c-Myc^+^ NP-Eα^−^ cells and that the former are poised to enter cell cycle.

Conversely, c-Myc-expressing LZ B cells with lower affinity BCRs showed greater expression of negative regulators of cell cycle entry *Cdkn1a* and *Id3* and signaling modifiers *Tbl1A*, *Cblb*, and *Trim56* (Figure S3I). This population also expressed more *Bach2*, which is inversely correlated with the strength of T cell help and positively correlated with memory B cell differentiation (Figures 4G and 4H)^24,25^. Consistent with these observations, c-Myc^+^ LZ B cells with lower affinity BCRs are enriched in expression of pre-memory associated transcription factors such as *Hhex*, *Mndal*, and *Tle3*^26^, memory-associated markers, including *Efnb1, Cd38*, and *Lifr*^27*–*29^, and the anti-apoptotic gene *Bcl2l1* (Figures 4G and S3J)^30^. CCR6 is reported to mark a population of pre-Memory cells in the LZ; however, we failed to detect enrichment of CCR6^+^ cells in the LZ B1-8^hi^ c-Myc^+^ NP-Eα^−^ population (Figures S4A–S4D)^31^. We conclude that B cells with lower affinity receptors that receive T cell help display features associated with the pre-memory compartment^24,26,32^.

To uncouple the effects of antigen capture and cognate Tfh interactions from BCR signaling, we normalized the amount of antigen presented by GC B cells in a BCR-independent manner using a chimeric antibody to deliver OVA antigen (αDEC-OVA)^6,33^. To validate that targeted antigen presentation among NP^+^ and NP^−^ cells is equivalent, we used αDEC-OVA-Eα to deliver Eα peptide (Figure S4E). Peptide presentation, as measured by Y-Ae staining, was indistinguishable between NP^+^ and NP^−^ cells (Figures S4E and S4F). After priming, we adoptively transferred a mixture of B1-8^hi^ c-Myc-GFP DEC205-sufficient and knockout B cells (B1-8^hi^ DEC205^−/−^) and injected αDEC-OVA to deliver OVA to DEC205-sufficient GC B cells, irrespective of their ability to bind antigen as measured by NP-Eα (Figure 4I). Under these conditions, the fraction of c-Myc^+^ LZ B cells was significantly higher among NP-Eα^+^ cells than their NP-Eα^−^ counterparts (Figure 4J). Thus, even when LZ B cells are loaded with similar amounts of antigen, irrespective of BCR affinity, selection is enriched among cells that demonstrably engage antigen, suggesting that selection signals are enhanced among cells that have also received strong BCR signals.

To examine the gene expression profiles of antigen-binding and nonbinding LZ B cells in the absence of detectable positive selection, we compared the transcriptomes of c-Myc^−^ cells. GSEA showed that c-Myc^−^ antigen-binding cells were enriched in pathways associated with BCR stimulation and activation and hallmark pathways indicative of metabolic changes (Figures 4K–4M, S4G, and S4H)^23,34^. The c-Myc reporter is limited in its sensitivity and may fail to report small changes in transcription. However, RNA-seq confirmed low *Myc* expression in GFP^−^ cells (Figure S4I). The magnitude of these metabolic changes is far smaller than those induced among positively selected GFP^+^ cells (Figures S4J and S4K). Together, these signatures suggest that in the relative absence of transcriptional signatures associated with positive selection (Figures S7I–S7K), LZ B cells that engage antigen signal through the BCR and activate metabolic pathways.

### GC BCR engagement protects LZ cells from apoptosis

**Figure 5.**
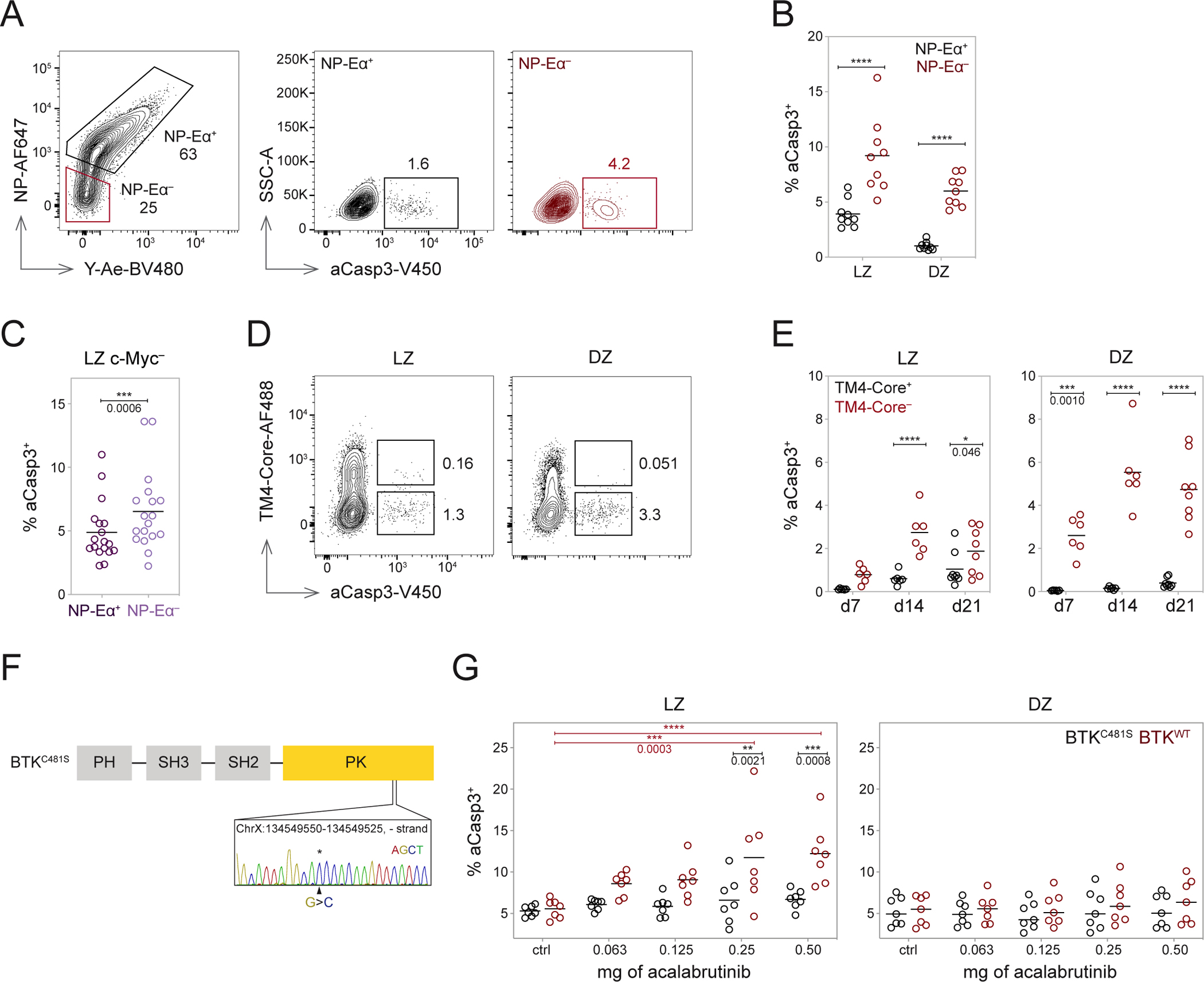
BCR engagement is necessary for B cell survival in the LZ. **(A)** Representative gating of aCasp3^+^ cells among NP-Eα^+^ and NP-Eα^−^ B1-8^hi^ cells. **(B)** Frequency of aCasp3^+^ cells among NP-Eα^+^ (black) and NP-Eα^−^ (red) cells labeled *in vivo* with ^14^NP-Eα, ****p<0.0001. **(C)** Frequency of aCasp3^+^ cells among c-Myc^−^ NP-Eα^+^ and c-Myc^−^ NP-Eα^−^ LZ B1-8^hi^ cells, two-tailed paired t test, ***p=0.0006. **(D)** Plots depicting aCasp3^+^ cells among TM4-core binding and nonbinding populations. **(E)** Frequency of aCasp3^+^ cells among TM4-core binding and nonbinding population over time, gated on LZ or DZ, then TM4-core^+^ or TM4-core^−^, *p=0.046 ***p=0.0010, ****p<0.0001. **(F)** Knock-in BTK^C481S^ point mutation. **(G)** Frequency of aCasp3^+^ cells among BTK^C481S^ and BTK^WT^ cells in the LZ (left) and DZ (right) with acalabrutinib treatment, two-way ANOVA with Šidák’s multiple comparisons (within dose) or Tukey’s multiple comparisons (across doses), ** and ***p values as marked, ****p<0.0001. Data from two (E and G), three (B), and five (C) independent experiments. Each dot represents one mouse, and lines depict means (B, C, E, and G). P values (B and E) calculated with RM two-way ANOVA with Šidák’s multiple comparisons. See also Figures S5.

To determine whether antigen binding confers a survival advantage to LZ B cells in the absence of positive selection, we measured cell death by apoptosis using activated caspase 3 expression (aCasp3) as a reporter^18^. Antigen-binding LZ and DZ B cells showed lower frequencies of aCasp3^+^ cells than their NP-Eα^−^ counterparts (Figures 5A and 5B). This effect was independent of selection because c-Myc^−^ LZ B cells that bound antigen were protected from apoptosis compared with lower affinity cells (Figure 5C). To determine whether a similar survival advantage is observed in a polyclonal immune response, we immunized mice with an HIV-1 antigen, TM4-Core^35^, and identified cells capable of antigen binding by flow cytometry using TM4-Core-AF488 (Figures 5D, 5E, and S5A). Polyclonal GC LZ B cells unable to bind TM4-Core-AF488 were significantly more likely to undergo apoptosis than antigen-binding cells (Figure 5E). We conclude that LZ B cells that engage antigen have a survival advantage.

### Continuous BCR signaling is necessary for LZ survival and positive selection.

To further examine the possibility that BCR signaling per se confers a survival advantage, we inhibited Bruton’s Tyrosine Kinase (BTK) with ibrutinib^36^. BTK is downstream of the BCR and required for tonic and antigen-dependent receptor signaling^37,38^. Moreover, *Btk* is not expressed in T cells^39^. When mice were treated with ibrutinib by subcutaneous injection, as little as 1.6 μg of ibrutinib was sufficient to induce a significant increase in frequencies of aCasp3^+^ LZ B cells one hour after injection (Figures S5B and S5C). Moreover, LZ B cells were significantly more sensitive to BTK inhibition than DZ B cells (Figure S5C).

To determine whether the effect of ibrutinib is B cell autonomous, we produced knock-in mice that carry a C481S mutation in BTK, which renders the enzyme insensitive to ibrutinib^40^ (Figure 5F). Development of BTK^C481S^ B cells was indistinguishable from wild-type counterparts in the bone marrow and the periphery. As expected, BTK^C481S^ B cells were resistant to ibrutinib-mediated inhibition of Ca++ flux upon BCR crosslinking (Figures S5D–S5F). Mixed bone marrow chimeras transplanted with BTK^C481S^ and BTK^WT^ cells were immunized with TM4-core and treated with acalabrutinib, a second-generation version of ibrutinib with improved specificity and reduced off-target binding to other Tec family kinases (Figures S5G–S5K)^41,42^. Whereas inhibitor treatment did not measurably increase apoptosis of DZ cells in either BTK^C481S^ or BTK^WT^ cells, BTK^WT^ LZ cells showed a significant dose-dependent increase in aCasp3^+^ staining (Figure 5G). The greatest BTK inhibition-induced cell death was seen at 2 hours, with rapid recovery by 12 hours (Figures S5L and S5M). We conclude that continuous BCR signaling is necessary for LZ B cell survival in the GC.

### BCR signaling synergizes with T cell help.

**Figure 6.**
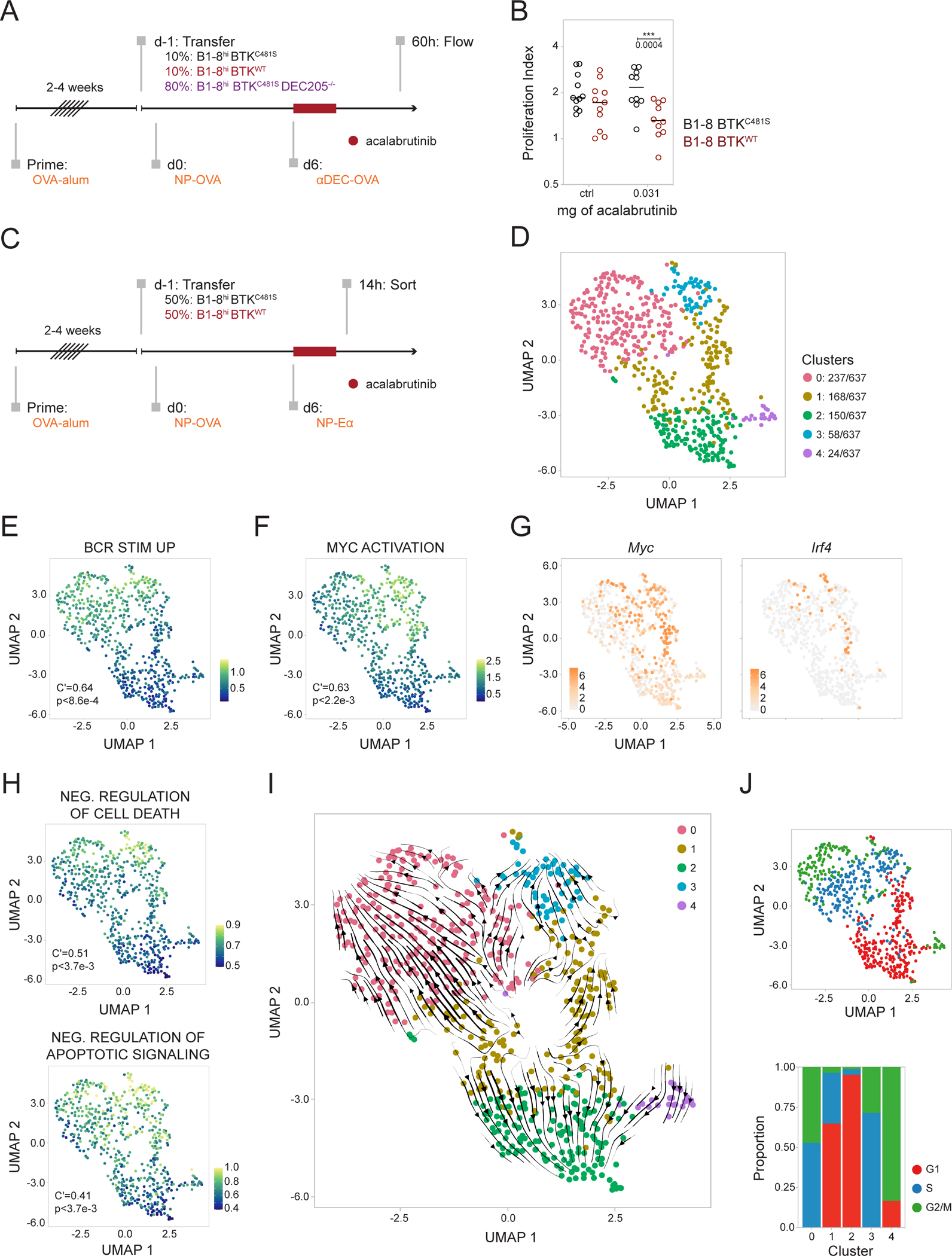
BCR signaling synergizes with T cell help. **(A)** Experimental setup. B1-8^hi^ BTK^C481S^, B1-8^hi^ BTK^WT^, and B1-8^hi^ BTK^C481S^ DEC205^−/−^ were transferred into OVA-primed hosts at the indicated ratios. Six days later, 5 μg of αDEC-OVA was injected (t=0h), and 0.03125 mg of acalabrutinib, or vehicle alone, was delivered by oral gavage at t=0h, 6h, and 12h. Readout by flow 60h after αDEC-OVA and dose 1 of drug (t=60h). **(B)** Proliferation index of B1-8^hi^ BTK^C481S^ and B1-8^hi^ BTK^WT^ 60h after αDEC-OVA treatment, calculated as a ratio of the frequency of population 60h after treatment with αDEC-OVA: with PBS, RM two-way ANOVA with Šidák’s multiple comparisons, ***p=0.0004. **(C)** Experimental setup. B1-8^hi^ BTK^C481S^ and B1-8^hi^ BTK^WT^ were transferred at indicated ratios. Mice were treated with 0.03125 mg of acalabrutinib, or vehicle alone, by oral gavage at t=0h, 6h, and 12h and sacrificed for sorting 2h after the last dose (t=14h). **(D)** Uniform manifold approximation and projection (UMAP) plot showing color-coded clustering of LZ cells. Number of cells/cluster indicated. **(E)** Enrichment of genes upregulated with stimulation through the IgG BCR^43^ and **(F)** of Myc activation pathway^44^, visualized on UMAP by signature scores. **(G)** Expression of *Myc* (left) and *Irf4* (right). **(H)** Enrichment of Gene Ontology gene signatures associated with the negative regulation of cell death (top) and anti-apoptotic signaling (bottom), visualized by signature scores. **(I)** Embedding of RNA velocity analysis onto UMAP. **(J)** Cell cycle phases visualized on UMAP and by cluster (bottom). Data representative of four independent experiments, each dot represents one mouse, and lines depict means (B). Autocorrelation and p values depicted on graphs (E, F, and H). See also Figures S6 and S7.

To examine the synergy between BCR signaling and T cell help, we adoptively transferred DEC205-sufficient drug-resistant (B1-8^hi^ BTK^C481S^) and drug-sensitive (B1-8^hi^ BTK^WT^) cells, and B1-8^hi^ drug-resistant DEC205-knockout (B1-8^hi^ BTK^C481S^ DEC205^−/−^) cells into OVA-primed mice and delivered antigen in a BCR-independent manner using αDEC-OVA (Figures 6A and S6A). Acalabrutinib was administered at a concentration that did not measurably alter survival (Figures S6B–S6E) or the relative frequency of wild-type and resistant LZ B cells prior to DZ migration (Figures S6F and S6G). Nevertheless, the proliferation of B1-8^hi^ BTK^WT^ drug-sensitive cells in the DZ 60 hours after αDEC-OVA delivery was significantly reduced compared to B1-8^hi^ BTK^C481S^ drug-resistant cells (Figure 6B and S6H). Thus, LZ BCR signaling synergizes with T cell help to determine the extent of DZ proliferation.

To investigate the mechanistic basis for synergy between LZ BCR signaling and T cell positive selection signals, we performed whole transcriptome single-cell RNA sequencing on drug-resistant B1-8^hi^ BTK^C481S^ and drug-sensitive B1-8^hi^ BTK^WT^ LZ GC B cells sorted from immunized mice treated with acalabrutinib or vehicle alone (Figures 6C and S7A). Cells were distributed across 5 clusters as visualized by Uniform Manifold Approximation and Projection (UMAP) (Figure 6D). We defined the clusters based on their top differentially expressed genes and their enrichment of gene signatures (Figures 6D and Table S1). Clusters 0 and 3 contain cycling cells and are enriched in both BCR signaling and positive selection gene programs^43,44^ (Figures 6E–6G, S7B, and S7C). A fraction of cells in cluster 3 co-express genes associated with plasma and pre-plasma cell fates, including *Xbp1*, *Irf4,* and *Prdm1*, suggesting that cluster 3 represents plasma and pre-plasma cells (Figures 6G and S7C)^45^. Cells in cluster 1 express the greatest amount of *Myc* transcript and are enriched in positive selection signatures, including Myc and mTOR pathways (Figures 6F, 6G, S7B, and S7C)^44,46–48^. Cells in cluster 2 show decreased expression of gene signatures associated with prevention of apoptosis and marked absence of BCR signaling (Figures 6E and 6H)^43^. Lastly, cells in cluster 4 are distinguished by their high expression of *Ccnb2* and resemble DZ-like cells that recently migrated to the LZ (Figures S7D, S7E, and Data S1)^6^.

To understand the dynamic relationships between the 5 clusters, we performed RNA velocity analysis and projected trajectories onto our UMAP (Figure 6I)^49,50^. Cells in cluster 4, which resemble recent LZ entrants, point towards Cluster 2, suggesting that cells that recently transitioned to the LZ resemble Cluster 2 cells in that they have not yet engaged their BCRs (Figures 6E and 6I). Cells in cluster 2 have trajectories pointing away from other clusters, which is consistent with the idea that LZ BCR signaling is required to promote transcriptional programs that enable subsequent GC B cell development and protect cells from apoptosis (Figures 6H and 6I). Trajectory analysis captures the cell cycle dynamics between clusters 0, 1, and 3, with streamlines pointing in the direction of cell cycle progression (Figures 6I and 6J)^51^. Cells in cluster 1 are bifurcated into one trajectory, right, pointing towards cluster 3, and another trajectory, left, towards cluster 0 (Figure 6I). Cells along this right axis are relatively enriched in Myc and mTOR activation pathways and show greater gene expression of *Cd40, Icam1,* and *Cd86*—which enhance stable conjugates with Tfh—and *Tnfrsf14* (Figures 6G, 6I, and S7C)^52,53^. These cells are also higher in their expression of *Myc*, *Batf*, and *Irf4*, suggesting that cells along this axis (right) receive a greater magnitude of T cell help, promoting their entry into cell cycle and differentiation into plasma cells (Figures 6G and S7C)^52,54,55^. Cells along the left axis pointing towards cluster 0 show increased expression of *Cxcr4* and *Polh* suggesting that they are poised to transition to the DZ (Figures S7D and S7E)^6^.

**Figure 7.**
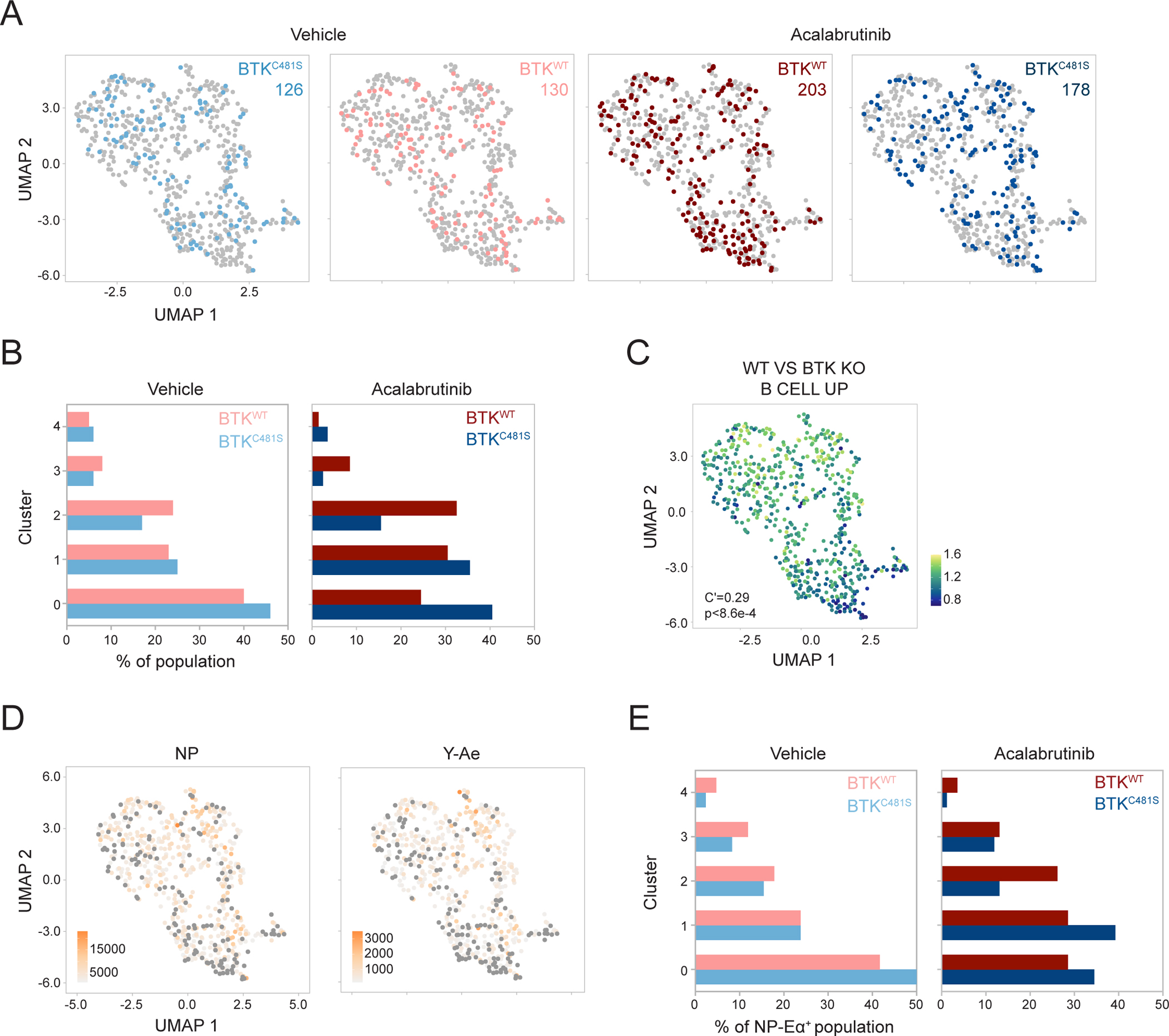
BCR signaling is a prerequisite to compete in the LZ. **(A)** Distribution of B1-8^hi^ BTK^C481S^ and B1-8^hi^ BTK^WT^ from mice treated with vehicle alone (far-left, left) or with acalabrutinib (right, far-right). Number of cells in condition denoted. **(B)** Cluster distribution of B1-8^hi^ BTK^C481S^ and B1-8^hi^ BTK^WT^ from mice treated with vehicle (left) and acalabrutinib (right). Frequencies calculated from a random sample of 100 cells from each population. **(C)** Enrichment of signature upregulated in WT vs BTK KO cells, visualized by signatures scores^56^. **(D)** NP (left) and Y-Ae (right) MFIs visualized on UMAP. Gray circles mark NP^−^ (left, MFIs <1000) and Y-Ae^−^ (right, MFIs <150) cells. **(E)** Cluster distribution of B1-8^hi^ BTK^C481S^ and B1-8^hi^ BTK^WT^ NP-Eα^+^ cells from mice treated with vehicle (left) or with acalabrutinib (right). Frequencies calculated from a random sample of 85 cells from each population. Autocorrelation and p value depicted on graph (C).

To determine how BCR signaling in the LZ impacts selection, we compared the distribution of acalabrutinib-sensitive, BTK^WT^, and -resistant, BTK^C481S^, cells in the presence or absence of inhibitor. In mice treated with acalabrutinib, BTK^WT^ cells are skewed towards cluster 2, which is characterized by a transcriptome that resembles BTK deletion (Figures 7A–7C)^56^, absence of BCR signaling, and lower expression of anti-apoptotic gene signatures (Figures 6E and 6H). The enrichment of BTK^WT^ cells in cluster 2 was present even among NP-Eα^+^ cells, suggesting that BTK inhibition prevents LZ cells binding and presenting antigen from progressing through the GC reaction (Figures 7D and 7E). Thus, whole transcriptome single-cell RNA sequencing indicates that LZ B cells that fail to signal through their BCRs remain in the G1 phase and undergo apoptosis.

## Discussion:

Current models for GC B cell selection posit that antibody affinity is selected indirectly when B cells with higher affinity receptors extract, process, and present antigen to Tfh^2,57^. Tfh play an essential role in this process by physically engaging with B cells through cell surface receptor-ligand interactions and secretion of cytokines^58–61^. Positive selection directs migration to the DZ, where selected cells undergo a proliferative burst proportional to the amount of antigen presented and the magnitude of T cell help^62^. The function of the BCR in this model is to act as an endocytic receptor for antigen capture^6^.

In addition to antigen capture, the BCR is also a signaling receptor and BCR stimulation has been shown to synergize with Tfh signals such as CD40^11,63^. However, GC B cells show attenuated BCR signaling due in part to increased SHP-1 and SHIP-1 phosphatase activity^7^. Additional alterations include poor signal propagation through protein kinase C-β, resulting in altered synapse formation and inefficient activation of NF-κB^9^. GC B cells also have elevated PTEN expression, which alters the ratio of secondary signaling messengers, redirecting the specificity of AKT, leading to the activation of negative regulators of BCR signaling^12^. These changes contributed to the initial suggestion that GC BCR signaling is silenced *in vivo*^7^. However, a Nur77-eGFP indicator mouse strain identified a population of LZ cells actively signaling through the BCR^8,64^.

We aimed to uncouple the dual functions of the BCR by introducing fluorescently labeled NP-Eα into GC reactions as a dynamic reporter of BCR engagement *in vivo* that confers no additional cognate antigen for presentation to OVA-specific Tfh. The small amounts of low-valency NP-Eα injected did not alter GC B cell survival, allowing direct examination of the role of BCR signaling in selection^65,66^. Combining NP-Eα with a c-Myc reporter revealed that LZ B cells with higher affinity receptors show higher expression of pathways associated with positive selection, even when cognate antigen presentation has been normalized in a BCR-independent manner. In contrast, c-Myc-expressing LZ B cells with lower affinity receptors showed higher gene expression of transcription factors BACH2 and HHEX^24,26^ and resemble a previously identified pre-Memory population^67^. Overall, these observations are consistent with the idea that memory B cells differentiate from lower affinity LZ GC B cells^24,25,31,68^.

A substantial fraction of LZ B cells shows reduced or undetectable affinity for antigen, indicating that an affinity-dependent checkpoint for DZ-LZ entry does not exist. However, LZ B cells with low-affinity receptors are more likely to die by apoptosis. BCR signaling is required to prevent apoptosis because inhibition of BTK specifically impacts LZ B cell survival. DZ cells are less sensitive to BTK inhibition, which may reflect the differential sensitivity of tonic signaling to ibrutinib treatment^69^. Upon binding to antigen, naïve B cells are inhibited by NR4A signaling and must receive a second activation signal within a short time window to avert mitochondrial dysfunction and apoptosis^70,71^. Whereas naïve B cells are sensitive to BCR activation-induced cell death in an avidity-dependent manner and sustained by tonic signaling^72,73^, our results suggest that LZ B cells are rewired such that tonic signaling is insufficient and they depend on antigen-derived signaling for their survival. Thus, survival in the LZ is dependent on BCR signaling and is not determined solely by the antigen capture function of the BCR and Tfh neglect^18,74^.

In addition to a survival advantage, BCR engagement in the LZ primes high-affinity B cells to receive positive selection, and trajectory analysis suggests that plasma cell differentiation appears to be favored among the LZ cells receiving the greatest amount of T cell help^52,54,63^. BCR stimulation *in vitro* activates metabolic pathways associated with transition to a state that amplifies co-stimulatory signals^71^. In the absence of detectable *Myc* expression, antigen-binding LZ cells showed similar metabolic changes, indicating that they are primed to amplify positive selection signals upon receiving T cell help. Furthermore, inhibiting BCR signaling directly affects the magnitude of DZ proliferation, even when cells are loaded with antigen in a BCR-independent manner. In conclusion, the data show that BCR signaling in the LZ facilitates positive selection by prolonging survival and by priming B cells to receive synergistic Tfh signals.

### Limitations of the study:

Tracking with NP-Eα is limited to a time window of around 24 hours and the signal is gradually lost. Therefore, it is not possible to follow multiple rounds of selection and post-GC cell fates. Although not shown, the NP-Eα tracker can be used to assess polyclonal responses, but whether a similar tracker can be constructed for other antigens is not explored in this study. We also do not address a possible role for priming and survival signals from FDCs.

The c-Myc-GFP reporter is limited in its sensitivity, and small changes in transcription may not be detectable as a GFP signal. However, when we examined the amount of *Myc* transcript among the c-Myc-GFP^−^ population in our bulk RNA-seq analysis, we detected only a small amount of transcript. Additional mechanistic experiments to understand the priming and synergistic nature of metabolic changes induced by BCR engagement would also be of interest for future studies.

## STAR Methods

### Resource Availability:

#### Lead Contact:

Additional information and requests for resources and reagents should be directed to and will be fulfilled by the lead contact, Michel C. Nussenzweig (nussen@rockefeller.edu).

#### Materials Availability:

Reagents, plasmids, and mouse lines reported in this study are available upon signing a Materials Transfer Agreement.

#### Data and Code Availability:

Bulk and single-cell RNA-seq data have been deposited at GEO and are publicly available as of the date of publication. Accession numbers are listed in the key resources table.

No original code has been reported in this paper.

Any additional information required to reanalyze the data reported in this paper is available from the lead contact upon request.

### Experimental Models and Subject Details:

#### Mice

Mice used in this study were group housed (up to 5 mice of the same sex) with unrestricted access to water and standard chow diet, unless otherwise indicated, under specific pathogen free conditions in the Rockefeller University (RU) Comparative Bioscience Center. Mice used in this study ranged from 6 to 14 weeks old. Wild-type C57BL/6J and B6.SJL male mice were purchased from Jackson Laboratories. B1-8^hi^, B1-8^hi^ DEC205^−/−^, B1-8^hi^ CFP, and B1-8^hi^ c-Myc-GFP have been described^6,20,75^. BTK^C481S^ point mutation mice were generated by microinjection of gRNA, hCas9, and single-stranded donor oligonucleotides into B6 zygote pronuclei (RU CRISPR and Genome Editing Center, RU Transgenic and Reproductive Technology Center)^76^. Mutants were backcrossed to B6.SJL for 5+ generations to remove possible CRISPR off-target effects. B1-8^hi^ BTK^C481S^ and B1-8^hi^ DEC205^−/−^ BTK^C481S^ were generated by crossing to B1-8^hi^ and B1-8^hi^ DEC205^−/−^. All experiments conform to protocols approved by the RU Institutional Animal Care and Use Committee.

### Method Details:

#### Bone Marrow Chimeras

Wild-type C57BL/6J or B6.SJL males, 6 weeks of age, were irradiated with two doses of 5 Gy each, with a resting period of 3–4 hours after the first dose. Donor bone marrow from littermate BTK^C481S^ or BTK^WT^ males was extracted by flushing tibias and femurs. Erythrocytes were lysed by resuspension in 1 mL of ACK buffer, and suspensions were filtered through a 70-μm filter. Single-cell suspensions were injected retro-orbitally into recipient mice following the second radiation dose. Mice were put on amoxicillin-laden chow for six weeks post-irradiation.

#### NP-Eα

4-Hydroxy-3-nitrophenylacetic acid Succinimide Ester (NP-Osu, Biosearch Technologies) was conjugated to Alexa Fluor 647 Streptavidin (SA-AF647) or Alexa Fluor 594 Streptavidin at a hapten:streptavidin molar ratio of 10:1 or 20:1. Biotinylated Eα_52–73_ peptide (N-biotin-GSGFAKFASFEAQGALANIAVDKA-COOH)^15,77^ was synthesized at the RU Proteomics Resource Center. NP-Streptavidin conjugates were incubated with a 6x molar excess of biotinylated Eα peptide, and excess peptide was removed by dialysis. Hapten-protein conjugation ratios were calculated by measuring the absorbance value at 430 nm. For αDEC-OVA-Eα experiments shown in Figures S4E and S4F, NP-SA-AF647 conjugates were incubated with a 30x molar excess of D-biotin and excess D-biotin was removed by dialysis.

#### B cell transfer

Resting B cells were isolated from spleen tissue of donor male or female mice. Spleens were passed through a 70-μm filter into complete RPMI media supplemented with Fetal Bovine Serum (FBS) (2% v/v) and 1M HEPES (1% v/v). Erythrocytes were lysed by resuspension in 1–2 mLs of ACK buffer. B cells were purified by negative selection using MACS CD43 beads (Miltenyi Biotec), following manufacturers’ instructions, and 2–5×10^6^ B cells were transferred by intravenous (i.v.) injection into recipient male hosts.

#### Immunization and treatments

Host C57BL/6J and B6.SJL mice, 6–8 weeks of age, were primed by intraperitoneal injection of 50 μg Ovalbumin (OVA) precipitated in Imject Alum at a 1:2 ratio as described^6^. Only males were used as hosts and mice in experimental groups were littermates. 2–4 weeks after priming, B cells were adoptively transferred as described. Host mice were boosted by subcutaneous (s.c.) injection of 25 μg ^17^NP-OVA in hind footpads one day later. Popliteal lymph nodes (LNs) were collected, and single-cell suspensions were labeled for flow cytometry seven days after the boost. When indicated, 2 μg NP-Eα, 5 μg of αDEC-OVA (in-house), or αDEC-CS (in-house) in 1x DPBS were injected into hind footpads^78^. 5 μg αDEC-OVA-Eα ( in-house) was injected s.c., as indicated, 4 hours prior to sacrifice. For sheep red blood cell (SRBC, Colorado Serum) immunizations, SRBCs were washed twice with 1x DPBS, quantified, and 5 × 10^6^ SRBCs were injected into hind footpads. For TM4-Core immunizations^35^, 3–5 μg of TM4-Core (in-house) was mixed with Alhydrogel (InvivoGen) adjuvant 2% at a 1:2 ratio and injected into hind footpads.

#### Flow Cytometry

Popliteal LNs were isolated and resuspended in 1x DPBS supplemented with 1% Bovine Serum Albumin (BSA) and EDTA [2mM final] (PBE). Single-cell suspensions were achieved by mechanical disruption of LNs with disposable micro-pestles. For staining of Eα presentation on MHC-II, suspensions were stained with Fc-block and Y-Ae-biotin for 30 minutes. Cells were washed and passed through a 100-μm filter before staining with surface antibodies and fluorescently-labeled streptavidin. For TM4-Core-AF488 staining, TM4-Core-biotin (in-house) was incubated with Alexa Fluor 488 Streptavidin for 30 minutes, covered, before addition to suspensions. For aCasp3 staining, suspensions were washed in 1x DPBS before resuspension in BD fixation/permeabilization solution. Cells were fixed on ice for 30 minutes, washed twice with 1x Perm buffer, and stained at 4C with aCasp3 antibodies in 1x Perm buffer for 45 minutes. Data were acquired on a BD FACSymphony instrument.

#### Multiphoton Imaging

Imaging was performed as described^79^ using an Olympus FV1000 upright microscope fitted with a 25X 1.05NA Plan water-immersion objective and a Mai-Tai DeepSee Ti-Sapphire laser. LNs were collected, cleaned of excess adipose tissue, and sandwiched between two coverslips adhered with vacuum grease for imaging. FDC networks were identified by i.v. injection of αCD35-AF488 24 hours prior to imaging. For tracking antigen localization and capture, 2 μg of NP-Eα-AF594 was injected s.c. into hind footpads 24 hours prior to imaging. Imaging was performed at λ=910 nm. CFP and AF488 fluorescence emissions were collected in two channels, using a pair of CFP (480/40 nm) and YFP (525/50 nm) filters separated by a 505-nm dichroic mirror, with AF488 appearing as positive in both channels. A third filter was used for AF594 emissions (605/70 nm).

#### Cell Sorting

Cell sorting for single-cell BCR sequencing and bulk RNA-seq was performed on a BD FACSAria II. Lysis buffer was made fresh prior to each sort by supplementing TCL buffer (Qiagen) with 1% β−mercaptoethanol (Sigma-Aldrich). For single-cell BCR sequencing, single B cells were sorted into 96-well plates containing 5 μL lysis buffer. For sorting of GC populations for bulk RNA-seq, up to 400 cells, from four independent experiments, were sorted into 25 μL of lysis buffer. For single cell RNA-seq, LZ B cells, from two independent experiments, were sorted into 96-well plates containing 5 μL of lysis buffer using a BD FACSymphony S6 sorter. Samples were centrifuged and flash-frozen on dry ice.

#### Single-Cell BCR sequencing

Single-cell RNA was purified using magnetic beads (RNAclean XP, Beckman Coulter). RNA was reverse transcribed to cDNA using oligodT primers and Maxima H- reverse transcriptase (Thermo Fisher Scientific). Heavy chains and lambda light chains were amplified separately using consensus V_H_ and V_L_ forward primers and reverse constant primers^80,81^. Well-specific 9-nucleotide barcodes were introduced via PCR to the 5’ end. Plate-specific indexing was introduced via PCR by adapting Illumina Nextera DNA index sequences. PCR products from individual plates were pooled and purified using magnetic beads (Ampure XP, Beckman Coulter). Plates were pooled at equal concentrations and sequenced with a 500-cycle reagent Nano kit v2 (Illumina) on the Illumina Miseq platform. Oligo sequences are provided in Table S2.

#### Bulk and single-cell RNA-Seq Library Preparation

RNA was purified using magnetic beads (RNAclean XP) and reverse transcribed to generate “template-switched” cDNA using oligodT primers, template switch oligo, and Maxima H- reverse transcriptase. Pre-amplification was performed using KAPA HIFI HotStart ReadyMix (Roche) as described^82–84^. Libraries were purified using magnetic beads (AmpureXP). Tagmentation and indexing of bulk RNA-seq libraries was performed using a Nextera XT DNA Library Prep kit and Nextera XT Index Kit v2 Set A (Illumina), following manufacturer’s instructions. Single-cell RNA-seq libraries were prepared using an Illumina DNA Prep kit and indexed with IDT for Illumina Index Sets (Illumina), following manufacturer’s instructions. Libraries were sequenced on an Illumina NovaSeq platform (RU Genomics Resource Center). Oligo sequences are provided in Table S2.

#### Fab Production

Heavy and Light chain eBlocks (IDT) were cloned into human Fab and lambda expression vectors by restriction cloning^80,81^. His_6_-tagged Fabs and lambda light chains were expressed by transient transfection in Expi293F cells (Thermo Fisher Scientific), and were purified using Ni Sepharose 6 Fast Flow resin (Cytiva).

#### Bio-layer Interferometry

Bio-layer interferometry measurements were performed using a ForteBio Octet Red96 (Sartorius). Monovalent binding assays were performed using High precision streptavidin biosensors (Sartorius), loaded with ^16^NIP-BSA-biotin (Biosearch Technologies) [5.86nM]. Fabs were diluted in 1x Kinetics Buffer (KB) (Sartorius) and assayed at 100, 50, and 25 nM. Ligand-coated biosensors were regenerated by short incubation in HCl (Sigma-Aldrich) buffer followed by neutralization in 1x KB. For avidity measurements, Anti-human Fab-CH1 biosensors (Sartorius) were loaded with Fabs diluted in 1x KB [100 nM] and assayed with either ^2^NP-BSA or ^9^NP-BSA (Biosearch Technologies) at 0.33 and 0.11 μM.

#### BTK Inhibition

Ibrutinib (S2680, Selleckchem) or acalabrutinib (HY-17600, MedChemExpress) were solubilized in DMSO (0.5 mg/L). Inhibitor solution was then dissolved in a solution of 10% (2-hydroxypropyl)-cyclodextrin (Sigma-Aldrich) in 1x DPBS. Mice were treated either by oral gavage (200 μL) or by injection into hind footpads (25 μL) as indicated. Assuming an average weight of 25 g/mouse, treatment with 1.56–25 μg of ibrutinib corresponds to approximately (0.062 mg/kg-1 mg/kg). For acalabrutinib treatments, 0.03125–0.25 mg of acalabrutinib corresponds to approximately 0.00125–0.02 mg/kg, respectively. Ibrutinib and acalabrutinb have a reported ED_50_ of 0.91mg/kg and 0.34 mg/kg, respectively^42^.

#### Ca++ Flux Assay

Spleen tissue from BTK^C481S^ and BTK^WT^ mice were passed through a 70-μm filter into complete RPMI media supplemented with FBS (2% v/v) and 1M HEPES (1% v/v). Erythrocytes were lysed by resuspension in 1–2 mLs of ACK buffer. B cell suspensions were purified by negative selection using MACS CD43 beads, quantified, and mixed at equal concentrations. B cells were resuspended to 10^7^ cells/mL in PBE with 1x PowerLoad Concentrate (Thermo Fisher Scientific) and Indo-1 AM [2 μM] (Thermo Fisher Scientific). Cells were incubated, protected from light, at 37C for 30 minutes. After loading, cells were washed 2x and 2×10^6^ cells were plated in a 96 well plate with ibrutinib (concentrations indicated) for 30 minutes at 37C. Cells were washed 2x with RPMI 1640 medium, no phenol red (Thermo Fisher Scientific), 1% BSA, and rested in RPMI buffer on ice with surface-staining antibodies for 30 minutes. Stimulation was performed by addition of biotinylated Goat-Anti-mouse IgM [20 μg/mL] followed by Streptavidin (Jackson ImmunoResearch) [40 μg/mL].

### Quantification and statistical analysis:

Details of statistics including the tests, exact value and unit of n, and definition of center, dispersion, and precision are indicated in figure legends. Quantification and statistical analyses were performed in GraphPad Prism (Version 9.4.0), unless otherwise detailed below and in figure legends. Graphs generated using Prism were edited for appearance using Adobe Illustrator. Flow cytometry analysis was performed in FlowJo v.10 software (BD). Significance was defined as *p<0.05, **p<0.01, ***p<0.001, ****p<0.0001.

#### BCR Sequence Analysis

Sequences were demultiplexed, paired using Panda-Seq^85^, and processed using FastX-toolkit. Sequences were submitted to IMGT^86^ for analysis of somatic mutations, light chain usage and rearrangements, and unproductive sequences. Unmutated B1-8^hi^ sequences were identified by CDR3 sequences and the number of mutations.

#### Bulk RNA-seq analysis

Transcript abundance was quantified using kallisto v0.44.0^87^ with GRCm38 transcriptome (Ensembl release 94), and subsequently summarized to the gene amount using the R package tximport^88^. Follicular B cell samples served as an initial quality check but were not included in subsequent analyses. Two paired LZ outlier samples were not included due to poor sequencing quality. Differential gene expression analysis was performed using DESeq2 v.1.24^89^. Pairwise comparisons of populations was performed by Gene Set Enrichment Analysis^90^ using C2: curated, C7: immunologic signatures, and H: hallmark gene sets from the Molecular signatures database (MSigDB)^23,91^. All enriched pathways had nominal p values<0.05 and FDR q values<0.25.

#### Single-cell RNA-seq Analysis

The gene count matrix was generated by aligning raw reads to the mouse genome (GRCm39 release 107) using STARsolo 2.7.10a, requiring a simple overlap with a gene region (Genefull)^92^. The matrix was fed into Seurat for analysis and filtering^93^. Cells with a mitochondrial proportion >5% and/or a feature count <200 were discarded. Cells were normalized and scaled with sctransform^94^. Uniform Manifold Approximation and Projection (UMAP) and clustering were performed by selecting the first ten principal components. Single-cell BCRs were assembled using TRUST4 v1.0.7^95^. Signature scores were calculated using VISION^96^ and gene sets from MSigDB^23,91^. For RNA velocity analysis, BAM files were processed using Velocyto v0.17.17^49^, analyzed using scVelo using a stochastic model of transcriptional dynamics^50^, and trajectories were plotted in our UMAP.

#### Bio-layer Interferometry Analysis

Analysis was performed in Octet^®^ Analysis Studio (Sartorius). Biosensors loaded with individual Fabs were used as references for subtraction of background signals. Affinities were determined by modeling binding using a 1:1, partial dissociation model. Quality of fit for all curves was determined by three criteria: visual examination, R^2^ values, and c^2^ values. Dissociation constants were reported only from curves that had R^2^ ≥0.97 and c^2^<0.5. Area under the curve calculations were performed in GraphPad Prism.

## Supplementary Material

Table S1 Table S1: Top 20 markers for each cluster, Related to Figures 6 and 7.

Table S2 Table S2: Oligo sequences used for single-cell BCR sequencing, bulk RNA-seq, and single-cell RNA-seq, Related to STAR Methods.

Supplemental material

## Figures and Tables

**Table:** 

REAGENT or RESOURCE	SOURCE	IDENTIFIER
**Antibodies**		
Rat Anti-Mouse Anti-CD35	Absolute Antibody	Cat#: Ab00238-7.1, RRID:AB_397114
PE Rabbit Anti-Mouse Active Caspase 3, Clone: C92 605	BD Biosciences	Cat# 550821, RRID:AB_393906
PE Rat Anti-Mouse CD184, Clone 2B11	BD Biosciences	Cat# 551966, RRID:AB_394305
Rat Anti-Mouse CD16/CD32 , Clone 2.4G2	BD Biosciences	Cat# 553142, RRID:AB_394657
PE-Cy7 Hamster Anti-Mouse CD95, Clone Jo2	BD Biosciences	Cat# 557653, RRID:AB_396768
V450 Rabbit Anti-Mouse Active Caspase-3, Clone C92-605	BD Biosciences	Cat# 560627, RRID:AB_1727415
BV421 Hamster Anti-Mouse CD95, Clone Jo2	BD Biosciences	Cat# 562633, RRID:AB_2737690
BV421 Rat Anti-Mouse CD184, Clone 2B11	BD Biosciences	Cat# 562738, RRID:AB_2737757
BV605 Rat Anti-Mouse CD45R/B220, Clone RA3-6B2	BD Biosciences	Cat# 563708, RRID:AB_2738383
BV421Mouse Anti-Mouse CD45.1, Clone A20	BD Biosciences	Cat# 563983, RRID:AB_2738523
BV480 Streptavidin	BD Biosciences	Cat# 564876, RRID:AB_2869619
BV480 Rat Anti-Mouse CD45R/B220, Clone RA3-6B2	BD Biosciences	Cat# 565631, RRID:AB_2739311
BV421 Rat Anti-Mouse CD205 (DEC-205), Clone V18-949	BD Biosciences	Cat# 566375, RRID:AB_2744323
BUV737 Anti-Mouse CD45.2, Clone 104	BD Biosciences	Cat# 612778, RRID:AB_2870107
BUV737 Mouse Anti-Mouse CD45.1, Clone A20	BD Biosciences	Cat# 612811, RRID:AB_2870136
BV711 Rat Anti-Mouse CD86, Clone GL1	BD Biosciences	Cat# 740688, RRID:AB_2734766
BUV496 Anti-Mouse CD38, Clone 90	BD Biosciences	Cat# 741090, RRID:AB_2916913
BUV395 Rat Anti-Mouse Ig, λ1, λ2 & λ3 Light Chain, Clone R26-46	BD Biosciences	Cat# 744529, RRID:AB_2742303
BV480 Mouse Anti-Mouse CD45.1, Clone A20	BD Biosciences	Cat# 746666, RRID:AB_2743938
BUV805 Rat Anti-Mouse CD45R/B220, Clone RA3-6B2	BD Biosciences	Cat# 748867, RRID:AB_2873270
HuCAL Fab-MH NEGATIVE CONTROL	Bio-Rad	Cat# HCA051, RRID:AB_915480
Alexa Fluor 488 Rat Anti-Mouse CD38, Clone 90	BioLegend	Cat# 102714, RRID:AB_528796
FITC Mouse Anti-Mouse CD45.2, Clone 104	BioLegend	Cat# 109806, RRID:AB_313443
APC Mouse Anti-Mouse CD45.2, Clone 104	BioLegend	Cat# 109814, RRID:AB_389211
Alexa Fluor 488 Mouse Anti-Mouse CD45.2, Clone 104	BioLegend	Cat# 109816, RRID:AB_492868
Brilliant Violet 785 Mouse Anti-CD45.2 Mouse, Clone: 104	BioLegend	Cat# 109839, RRID:AB_2562604
PE Mouse Anti-Mouse CD45.1, Clone A20	BioLegend	Cat# 110708, RRID:AB_313497
Alexa Fluor 700 Mouse Anti-Mouse CD45.1, Clone: A20	BioLegend	Cat# 110724, RRID:AB_493733
APC Rat Anti-Mouse CD205, Clone: NLDC-145	BioLegend	Cat# 138206, RRID:AB_10613641
Biotin-SP (long spacer) AffiniPure Goat Anti-Mouse IgM, μ chain specific	Jackson Immunoresearch	Code: 115-065-020, RRID: AB_2338560
Recombinant αDEC-OVA	Produced In house	N/A
Recombinant αDEC-CS	Produced In house	N/A
Recombinant αDEC-OVA-Eα	Produced In house	N/A
PE Rat Anti-Mouse CCR6 Monoclonal antibody, Clone 140706	R and D Systems	Cat# FAB590P, RRID:AB_2244251
Biotin Mouse Anti-Mouse Ea52-68 peptide bound to I-A Mouse, Clone Yae	Thermo Fisher Scientific	Cat# 13-5741-85, RRID:AB_657823
APC-eFluor 780, eBioscience Anti-Mouse CD4, Clone RM4-5	Thermo Fisher Scientific	Cat# 47-0042-82, RRID:AB_1272183
APC-eFluor 780, eBioscience Anti-Mouse CD8a, Clone 53-6.7	Thermo Fisher Scientific	Cat# 47-0081-82, RRID:AB_1272185
APC-eFluor 780, eBioscience Anti-Mouse F4/80, Clone BM8	Thermo Fisher Scientific	Cat# 47-4801-82, RRID:AB_2735036
APC-eFluor 780, eBioscience Anti-Mouse Tcr beta, Clone H57-597	Thermo Fisher Scientific	Cat# 47-5961-82, RRID:AB_1272173
Alexa Fluor 700, eBioscience Anti-Mouse CD38, Clone 90	Thermo Fisher Scientific	Cat# 56-0381-82, RRID:AB_657740
**Chemicals, peptides, and recombinant proteins**		
Cytofix/Cytoperm Fixation/Permeabilization Solution Kit	BD Biosciences	Cat# 554714
NP-OVAL (Ovalbumin) Conjugation Ratio 17	Biosearch Technologies	Item ID N-5051-10
NP-OSu	Biosearch Technologies	Item ID N-1010-100
NIP-BSA-Biotin, Conjugation Ratio 16	Biosearch Technologies	Item ID N-1027-5
NP-BSA-Biotin, Conjugation Ratio 2	Biosearch Technologies	Item ID N-1026-5
NP-BSA-Biotin, Conjugation Ratio 9	Biosearch Technologies	Item ID N-1026-5
Alhydrogel^®^ adjuvant 2%	InvivoGen	Cat# vac-alu-250
Acalabrutinib	MedChem Express	Cat# HY-17600
Biotinylated Eα peptide	Nussenzweig Lab	N/A
Buffer TCL	Qiagen	Cat# 1031576
Ibrutinib, Free Base	Selleckchem	Cat# S2680
2-Mercaptoethanol,BioUltra, for molecular biology, ≥99.0% (GC)	Sigma-Aldrich	SKU 63689; CAS 60-24-2
Igepal^®^ CA-630,for molecular biology	Sigma-Aldrich	SKU I8896; CAS 9002-93-1
N,N-Dimethylformamide,anhydrous, 99.8%	Sigma-Aldrich	SKU 227056; CAS 68-12-2
(2-Hydroxypropyl)-beta-cyclodextrin,powder, BioReagent, suitable for cell culture	Sigma-Aldrich	SKU C0926; CAS 128446-35-5
Betaine solution, 5 M, PCR Reagent	Sigma-Aldrich	SKU B0300; CAS 107-43-7
Dimethyl sulfoxide	Sigma-Aldrich	SKU D2438; CAS 67-68-5
Albumin from chicken egg white, lyophilized powder, ≥98% (agarose gel electrophoresis)	Sigma-Aldrich	SKU A5503; CAS 9006-59-1
Hydrochloric acid solution	Sigma-Aldrich	SKU H9892; CAS 7647-01-0
Bovine Serum Albumin	Sigma-Aldrich	SKU A2153; CAS 9048-46-8
Fetal Bovine Serum	Sigma-Aldrich	SKU F8192; MDL MFCD00132239
ACK Buffer	Thermo Fisher Scientific	Cat# A1049201
RPMI 1640 Medium, no phenol red	Thermo Fisher Scientific	Cat# 11835030
DPBS, no calcium, no magnesium	Thermo Fisher Scientific	Cat# 14190144
Thermo Scientific Imject Alum Adjuvant	Thermo Fisher Scientific	Cat# 77161
RPMI Buffer	Thermo Fisher Scientific	Cat#11875093
Streptavidin, Alexa Fluor^™^ 647 conjugate	Thermo Fisher Scientific	Cat# S21374
Streptavidin, Alexa Fluor^™^ 594 conjugate	Thermo Fisher Scientific	Cat# S11227
Streptavidin, Alexa Fluor^™^ 488 conjugate	Thermo Fisher Scientific	Cat# S11223
Life Technologies Powerload Concentrate 100x	Thermo Fisher Scientific	Cat# P10020
Probenecid, Water Soluble	Thermo Fisher Scientific	Cat# P36400
HEPES	Thermo Fisher Scientific	Cat# 15630080
Indo-1 AM, cell permeant	Thermo Fisher Scientific	Cat# I1223
Powerload Concentrate 100x	Thermo Fisher Scientific	Cat# P10020
D-Biotin	Thermo Fisher Scientific	Cat# B20656
**Critical Commercial Reagents**		
Agencourt RNAClean XP, 40 mL	Beckman Coulter	Product# A63987
Agencourt AMPure XP	Beckman Coulter	Product# A63881
Sheep Red Blood Cells	Colorado Serum Company	Cat# 31112
Ni Sepharose 6 Fast Flow, 100 mL	Cytiva	Product# 17531802
Nextera XT DNA Library Preparation Kit	Illumina	Cat# FC-131-1096
Nextera XT Index Kit v2 Set A	Illumina	Cat# FC-131-2001
Illumina DNA Prep, (M) Tagmentation	Illumina	Cat# 20060059
IDT^®^ for Illumina^®^ DNA/RNA UD Indexes Set A	Illumina	Cat# 20027213
IDT^®^ for Illumina^®^ DNA/RNA UD Indexes Set B	Illumina	Cat# 20027214
IDT^®^ for Illumina^®^ DNA/RNA UD Indexes Set C	Illumina	Cat# 20027215
IDT^®^ for Illumina^®^ DNA/RNA UD Indexes Set D	Illumina	Cat# 20027216
Feeding Tube 20ga 38mm 250 Pk	Instech Laboratories	Part# FTP2038
Streptavidin, Unconjugated	Jackson ImmunoResearch	Code# 016-000-113
CD43 (Ly-48) MicroBeads, mouse	Miltenyi Biotec	Order# 130-049-801
Anti-Ter-119 MicroBeads, mouse	Miltenyi Biotec	Order# 130-049-901
LS Columns	Miltenyi Biotec	Order# 130-042-401
Pre-separation Filters	Miltenyi Biotec	Order# 130-041-407
NEB^®^ 5-alpha Competent *E. coli* (High Efficiency)	New England Biolabs	Cat# C2987I
Quick Ligation^™^ Kit	New England Biolabs	Cat# M2200L
Quick CIP	New England Biolabs	Cat# M0525L
AgeI-HF^®^ Restriction Enzyme	New England Biolabs	Cat# R3552L
SalI-HF	New England Biolabs	Cat# R3138L
XhoI	New England Biolabs	Cat# R0146L
QIAquick PCR & Gel Cleanup Kit	Qiagen	Cat# 28506
KAPA HiFi HotStart ReadyMix	Roche	Material# 7958935001
Kinetics Buffer 10X	Sartorius	Item# 18-1105
High precision streptavidin (SAX)	Sartorius	Item# 18-5119
Anti-Human Fab-CH1 (FAB2G)	Sartorius	Item# 18-5127
Pierce^™^ Slide-A-Lyzer^®^ MINI Dialysis Units, MWCO=10K	Thermo Fisher Scientific	Cat# 88404
Pierce^™^ Protein Concentrators, MWCO=10 kD	Thermo Fisher Scientific	Cat# 88513
Maxima H Minus Reverse Transcriptase	Thermo Fisher Scientific	Cat# EP0753
Thermo Scientific Adhesive PCR Plate Seals	Thermo Fisher Scientific	Cat# AB-0626
Qubit^™^ dsDNA HS and BR Assay Kits	Thermo Fisher Scientific	Cat# Q32854
Qubit^™^ 1X dsDNA High Sensitivity (HS) and Broad Range (BR) Assay Kits	Thermo Fisher Scientific	Cat# Q33231
**Deposited Data**		
Bulk-RNA seq (GC B1-8hi cells, Myc and NP-Ea)	this paper	GSE225573
sc-RNA seq (LZ B1-8hi BTK^C481S^ and LZ B1-8hi BTK^WT^)	this paper	GSE225574
**Experimental models: Cell Lines**		
Expi293F	Thermo Fisher Scientific	Cat# A14527, RRID:CVCL_D615
**Experimental models: Organisms/strains**		
B1-8^hi^	Nussenzweig Lab	N/A
BTK^C481S^	Nussenzweig Lab	N/A
DEC205^−/−^	Nussenzweig Lab	N/A
B6;129-*Myc*^*tm1Slek*/J^	The Jackson Laboratory	Strain #:021935, RRID:IMSR_JAX:021935
B6.SJL-*Ptprc*^*a*^ *Pepc*^*b*^/BoyJ	The Jackson Laboratory	Strain #:002014, RRID:IMSR_JAX:002014
C57BL/6-Tg(Nr4a1-EGFP/cre)820Khog/J	The Jackson Laboratory	Strain #:016617, RRID:IMSR_JAX:016617
C57BL/6J	The Jackson Laboratory	Strain #:000664, RRID:IMSR_JAX:000664
Tg(CAG-ECFP)CK6Nagy/J	The Jackson Laboratory	Strain #:003773, RRID:IMSR_JAX:003773
**Oligonucleotides**		
See Table S2		
**Software and algorithms**		
Velocyto	Bergen et al.^50^	http://velocyto.org/
scVelo	Kowalczyk et al.^51^	https://scvelo.readthedocs.io/
PANDAseq	Masella et al.^85^	https://github.com/neufeld/pandaseq
kallisto	Bray et al.^87^	https://pachterlab.github.io/kallisto/about
tximport	Soneson et al.^88^	https://bioconductor.org/packages/release/bioc/html/tximport.html
DESeq2	Love et al.^89^	https://bioconductor.org/packages/release/bioc/html/DESeq2.html
Gene Set Enrichment Analysis	Subramanian et al.^90^	https://www.gsea-msigdb.org/gsea/index.jsp
STARsolo	Kaminow et al.^92^	https://github.com/alexdobin/STAR
Seurat	Hao et al.^93^	https://satijalab.org/seurat/
sctransform	Choudhary et al.^94^	https://satijalab.org/seurat/index.html
TRUST4	Song et al.^95^	https://github.com/liulab-dfci/TRUST4/releases
VISION	DeTomaso et al.^96^	https://yoseflab.github.io/VISION/
R	N/A	https://www.r-project.org/
GraphPad Prism	N/A	https://www.graphpad.com/
FlowJo	N/A	https://www.flowjo.com/
Octet^®^ Analysis Studio	N/A	https://www.sartorius.com/en
FastX-toolkit	N/A	http://hannonlab.cshl.edu/fastx_toolkit/
Adobe Illustrator	N/A	https://www.adobe.com/

## Highlights

Developed a tracker of *in vivo* antigen binding and presentation.

Generated a Bruton’s Tyrosine Kinase drug-resistant mouse model.

BCR signaling protects LZ B cells from apoptosis.

BCR signaling primes and synergizes with T cell help.

## Inclusion and Diversity:

We support inclusive, diverse, and equitable conduct of research.
