# Supplementary material for: B cell receptor signaling in germinal centers prolongs survival and primes B cells for selection
Source: Immunity. Author manuscript; Available in PMC 2023 Aug 14. (PMC10424567; doi:10.1016/j.immuni.2023.02.003)
Supplement: Supplemental material [file NIHMS1882216-supplement-Supplemental_material.pdf]

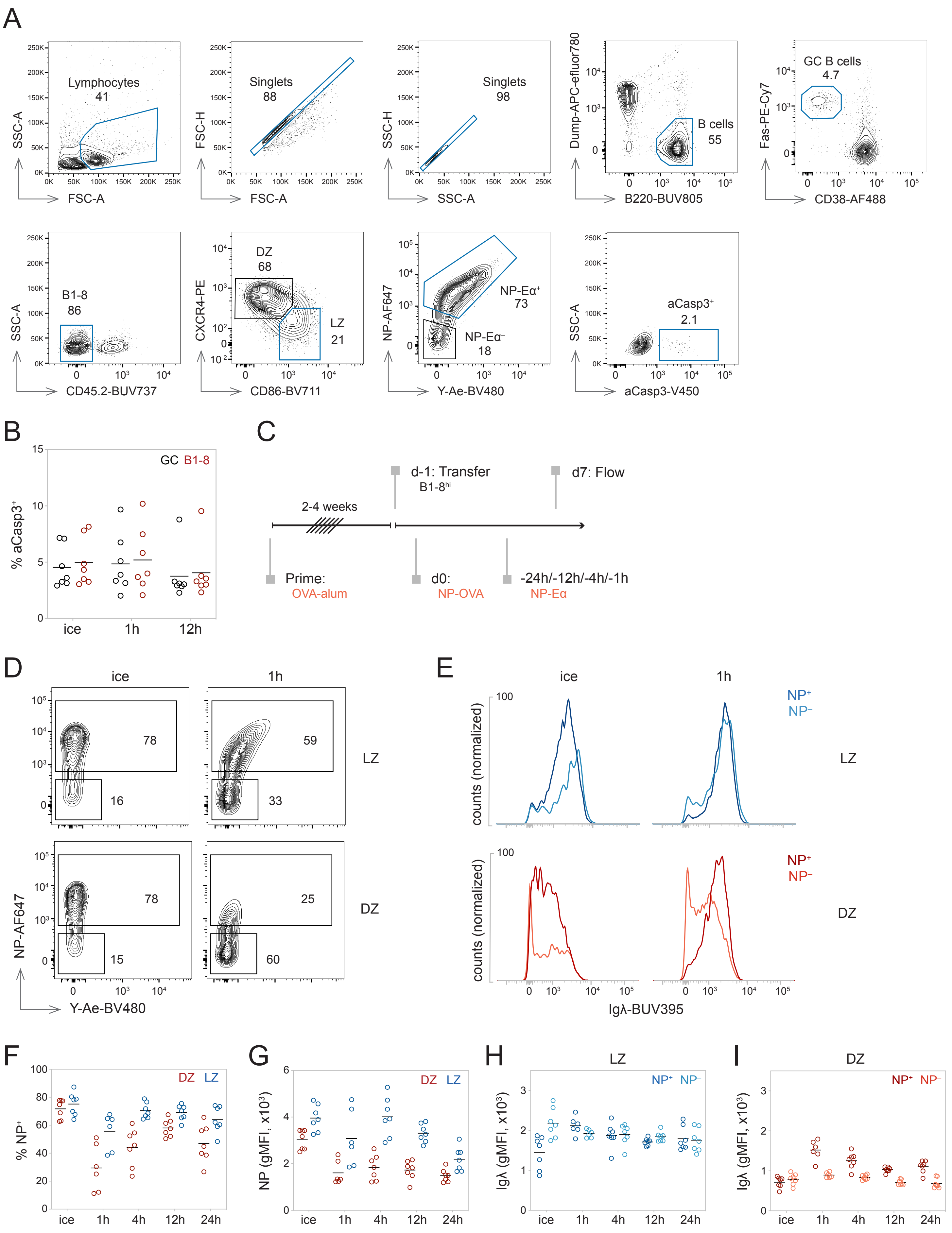
A
Lymphocytes
41
SSC-A
FSC-A
Singlets
88
FSC-H
FSC-A
Singlets
98
SSC-H
SSC-A
Dump-APC-efluor780
B cells
55
B220-BUV805
GC B cells
4.7
Fas-PE-Cy7
CD38-AF488
B1-8
86
SSC-A
CD45.2-BUV737
DZ
68
LZ
21
CXCR4-PE
CD86-BV711
NP-Eα+
73
NP-Eα−
18
NP-AF647
Y-Ae-BV480
aCasp3+
2.1
SSC-A
aCasp3-V450
B
GC B1-8
% aCasp3+
ice
1h
12h
C
2-4 weeks
d-1: Transfer
B1-8hi
d7: Flow
Prime:
OVA-alum
d0:
NP-OVA
-24h/-12h/-4h/-1h
NP-Eα
D
ice
1h
78
16
59
33
LZ
78
15
25
60
DZ
NP-AF647
Y-Ae-BV480
E
ice
1h
NP+
NP−
LZ
counts (normalized)
NP+
NP−
DZ
counts (normalized)
Igλ-BUV395
F
DZ LZ
% NP+
ice
1h
4h
12h
24h
G
DZ LZ
NP (gMFI, x10³)
ice
1h
4h
12h
24h
H
LZ
NP+ NP−
Igλ (gMFI, x10³)
ice
1h
4h
12h
24h
I
DZ
NP+ NP−
Igλ (gMFI, x10³)
ice
1h
4h
12h
24h

Figure S1. Kinetics and dynamic range of NP-Eα tracking in vivo, Related to Figure 1.

(A) Gating strategy for experimental setup, see Figure 1B.
(B) Frequency of aCasp3^{+} among total GC (black) or GC B1-8^{hi} (red) populations after NP-Eα injection, two-way ANOVA with Šidák's multiple comparisons, ns.
(C) Experimental setup. NP-Eα was injected 1 hour (h), 4 h, 12 h, or 24 h before sacrifice. Control mice were injected with SA-Eα and cells were labeled with NP-Eα on ice.
(D) Representative flow cytometry plots showing LZ (top) and DZ (bottom) B1-8^{hi} uptake and presentation of NP-Eα, ice and 1 h labeling shown.
(E) Representative histograms showing Igλ surface expression of LZ (top) and DZ (bottom) B1-8^{hi} NP^{+} and NP^{-} populations, ice and 1 h labeling shown.
(F) Summary of (D), frequency of NP^{+} DZ and LZ B1-8^{hi} over time.
(G) gMFI of NP-AF647 in NP^{+} DZ and LZ cells over time.
(H) Summary of (E), Igλ gMFI of LZ NP^{+} and NP^{-} populations over time.
(I) Summary of (E), Igλ gMFI of DZ NP^{+} and NP^{-} populations over time.
(B, F-I) Data from two independent experiments, each dot represents one mouse, and lines depict mean.

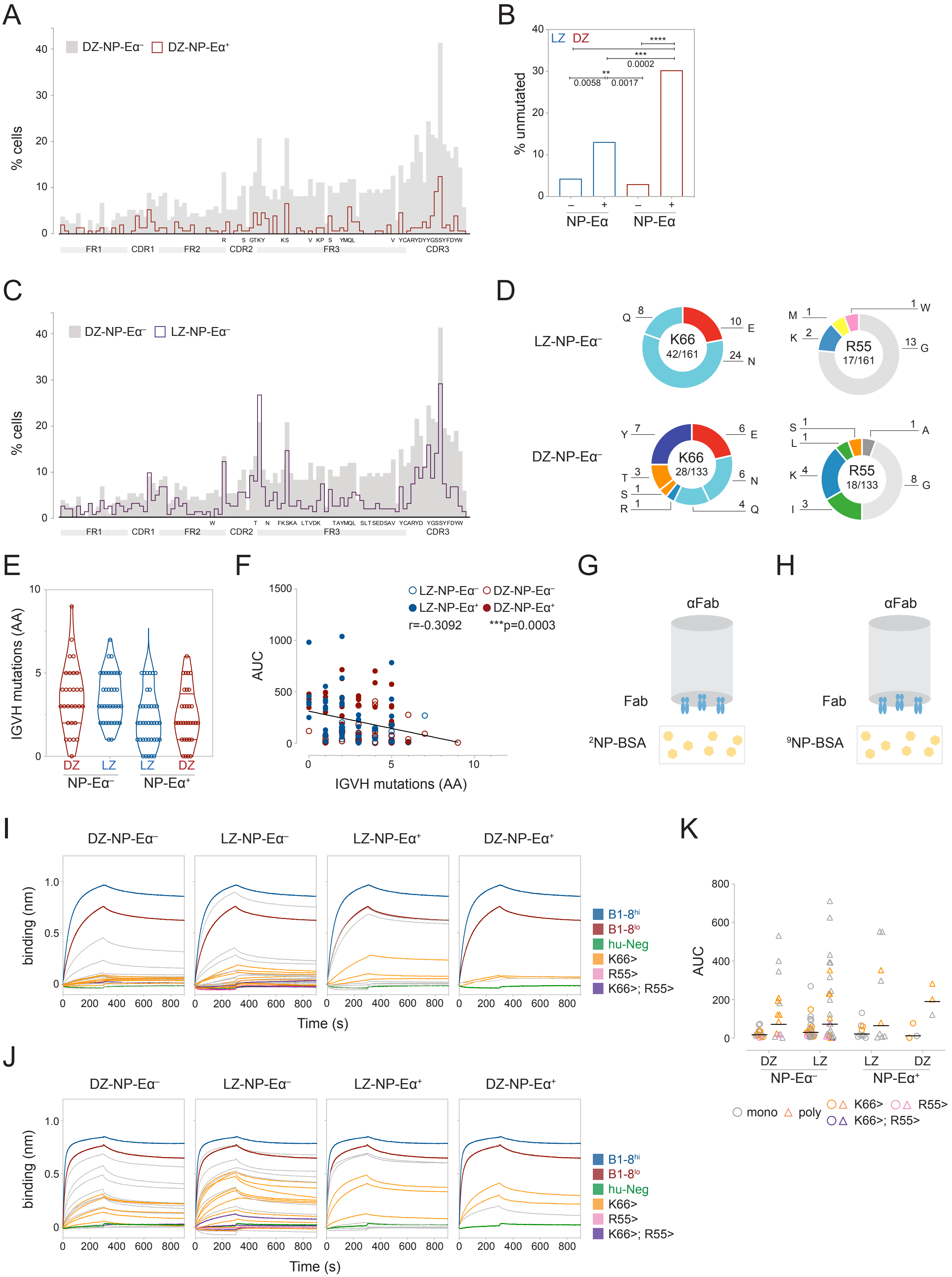

A
DZ-NP-Eα⁻
DZ-NP-Eα⁺
% cells
FR1
CDR1
FR2
CDR2
FR3
CDR3
B
LZ DZ
% unmutated
0.0058
0.0017
0.0002
NP-Eα
NP-Eα
C
DZ-NP-Eα⁻
LZ-NP-Eα⁻
% cells
D
LZ-NP-Eα⁻
DZ-NP-Eα⁻
K66
42/161
R55
17/161
K66
28/133
R55
18/133
E
IGVH mutations (AA)
DZ
LZ
LZ
DZ
NP-Eα⁻
NP-Eα⁺
F
LZ-NP-Eα⁻
DZ-NP-Eα⁻
LZ-NP-Eα⁺
DZ-NP-Eα⁺
r=-0.3092
***p=0.0003
AUC
IGVH mutations (AA)
G
αFab
Fab
²NP-BSA
H
αFab
Fab
⁹NP-BSA
I
DZ-NP-Eα⁻
LZ-NP-Eα⁻
LZ-NP-Eα⁺
DZ-NP-Eα⁺
binding (nm)
Time (s)
B1-8hi
B1-8lo
hu-Neg
K66>
R55>
K66>; R55>
J
DZ-NP-Eα⁻
LZ-NP-Eα⁻
LZ-NP-Eα⁺
DZ-NP-Eα⁺
binding (nm)
Time (s)
B1-8hi
B1-8lo
hu-Neg
K66>
R55>
K66>; R55>
K
AUC
DZ
LZ
LZ
DZ
NP-Eα⁻
NP-Eα⁺
mono
poly
K66>
R55>
K66>; R55>

Figure S2. Somatic Hypermutation and binding analyses of NP-Eα^+ and NP-Eα^- GC compartments, Related to Figures 2 and 3.

(A) Overlapping histograms depicting distribution of mutations among DZ-NP-Eα^- and DZ-NP-Eα^+ populations. Targeted AA residues found in >=5% of DZ-NP-Eα^- over DZ-NP-Eα^+ population are listed below axis.
(B) Fraction of unmutated cells in sorted populations, Fisher's exact test, ** and *** p values indicated, ****$p<0.0001$.
(C) Distribution of mutations in DZ-NP-Eα^- and LZ-NP-Eα^- populations. Targeted AA residues found in >=5% of DZ-NP-Eα^- over LZ-NP-Eα^- population are listed below axis.
(D) Targeted K66 and R55 residues in LZ-NP-Eα^- and DZ-NP-Eα^- cells and their AA replacements. Fraction shown in middle of plot denotes number of sequences with mutation over the number of sequences in the compartment.
(E) Number of AA mutations in IGVH chains of Fabs produced from LZ- and DZ-NP-Eα^+ and -NP-Eα^- compartments. Violin plot depicts median and quartiles.
(F) Scatterplot showing correlation between number of IGVH mutations and AUC from monovalent BLI setup, Spearman correlation, r=-0.3092, ***p=0.0003. Line depicts simple linear regression calculated from scatterplot (Y = -33.48*X + 311.1).
(G) Low polyvalent BLI setup. Individual Fabs are immobilized to anti-human Fab sensors with ^{2}NP-BSA [0.33 μM] in solution.
(H) High polyvalent BLI setup. Individual Fabs are immobilized to anti-human Fab sensors with ^{9}NP-BSA [0.11 μM] in solution.
(I-J) BLI traces of Fabs with no detectable monovalent binding from Figure 3 assayed with ^{2}NP-BSA or (J) with ^{9}NP-BSA.
(K) Summary AUC comparison of Fabs that showed undetectable monovalent binding (circles) assayed under polyvalent condition with ^{9}NP-BSA (triangles) (Figures 3, S2H and S2J).
Each dot represents one Fab (E, F, and K). Lines depicts geometric mean (K).

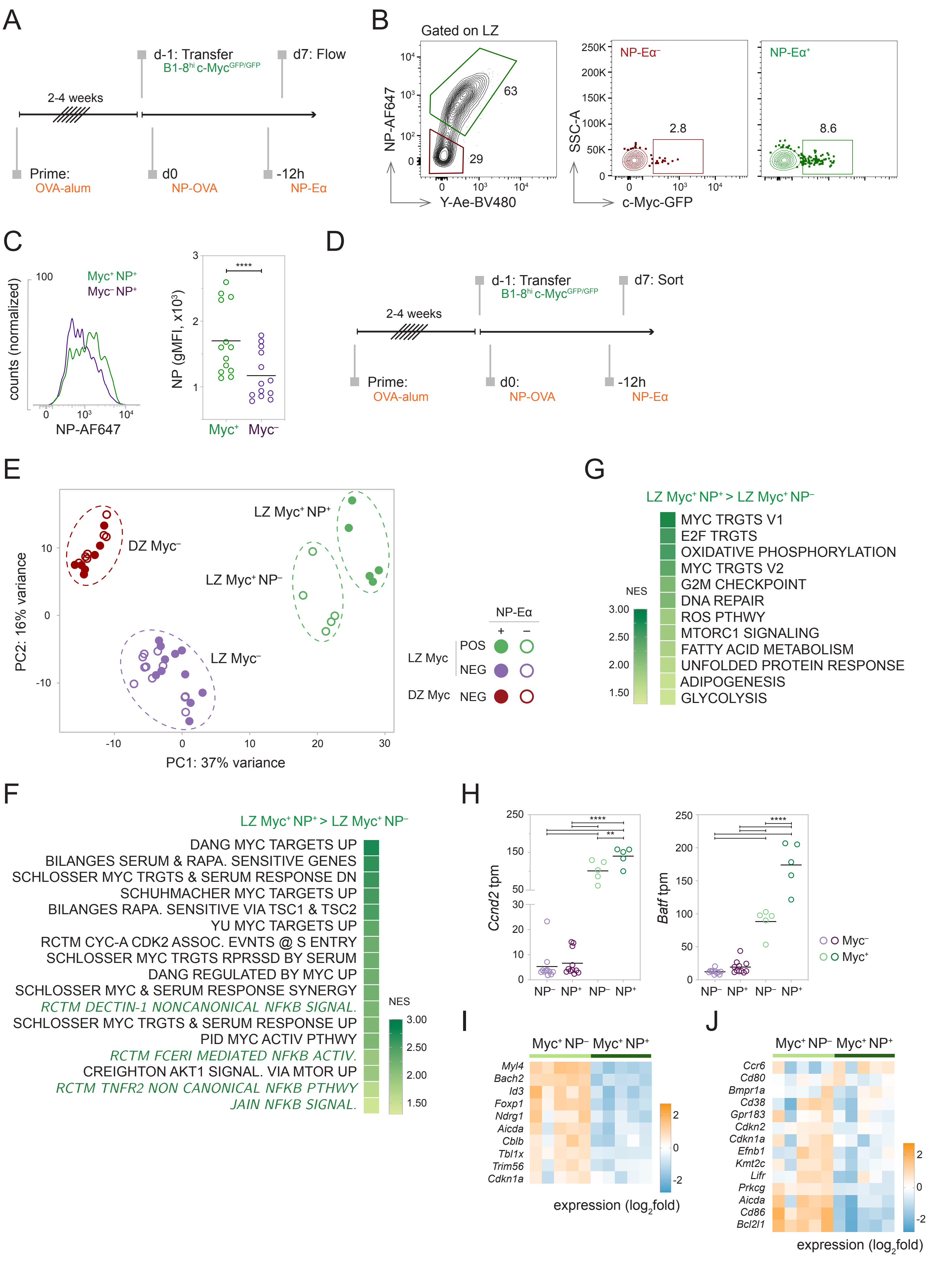

A
d-1: Transfer
B1-8hi c-MycGFP/GFP
d7: Flow
2-4 weeks
Prime:
OVA-alum
d0
NP-OVA
-12h
NP-Eα
B
Gated on LZ
NP-AF647
Y-Ae-BV480
63
29
NP-Eα⁻
NP-Eα⁺
2.8
8.6
SSC-A
c-Myc-GFP
C
counts (normalized)
Myc⁺ NP⁺
Myc⁻ NP⁺
NP-AF647
NP (gMFI, x10³)
****
Myc⁺
Myc⁻
D
d-1: Transfer
B1-8hi c-MycGFP/GFP
d7: Sort
2-4 weeks
Prime:
OVA-alum
d0:
NP-OVA
-12h
NP-Eα
E
DZ Myc⁻
LZ Myc⁺ NP⁺
LZ Myc⁺ NP⁻
LZ Myc⁻
PC2: 16% variance
PC1: 37% variance
NP-Eα
LZ Myc
POS
NEG
DZ Myc
NEG
G
LZ Myc⁺ NP⁺ > LZ Myc⁺ NP⁻
NES
MYC TRGTS V1
E2F TRGTS
OXIDATIVE PHOSPHORYLATION
MYC TRGTS V2
G2M CHECKPOINT
DNA REPAIR
ROS PTHWY
MTORC1 SIGNALING
FATTY ACID METABOLISM
UNFOLDED PROTEIN RESPONSE
ADIPOGENESIS
GLYCOLYSIS
F
LZ Myc⁺ NP⁺ > LZ Myc⁺ NP⁻
DANG MYC TARGETS UP
BILANGES SERUM & RAPA. SENSITIVE GENES
SCHLOSSER MYC TRGTS & SERUM RESPONSE DN
SCHUHMACHER MYC TARGETS UP
BILANGES RAPA. SENSITIVE VIA TSC1 & TSC2
YU MYC TARGETS UP
RCTM CYC-A CDK2 ASSOC. EVNTS @ S ENTRY
SCHLOSSER MYC TRGTS RPRSSD BY SERUM
DANG REGULATED BY MYC UP
SCHLOSSER MYC & SERUM RESPONSE SYNERGY
RCTM DECTIN-1 NONCANONICAL NFKB SIGNAL.
SCHLOSSER MYC TRGTS & SERUM RESPONSE UP
PID MYC ACTIV PTHWY
RCTM FCERI MEDIATED NFKB ACTIV.
CREIGHTON AKT1 SIGNAL. VIA MTOR UP
RCTM TNFR2 NON CANONICAL NFKB PTHWY
JAIN NFKB SIGNAL.
NES
H
Ccnd2 tpm
Batf tpm
****
**
NP⁻ NP⁺ NP⁻ NP⁺
Myc⁻
Myc⁺
I
Myc⁺ NP⁻ Myc⁺ NP⁺
Myl4
Bach2
Id3
Foxp1
Ndrg1
Aicda
Cblb
Tbl1x
Trim56
Cdkn1a
expression (log2fold)
J
Myc⁺ NP⁻ Myc⁺ NP⁺
Ccr6
Cd80
Bmpr1a
Cd38
Gpr183
Cdkn2
Cdkn1a
Efnb1
Kmt2c
Lifr
Prkcg
Aicda
Cd86
Bcl2l1
expression (log2fold)

Figure S3. Gene expression analysis and characterization of c-Myc^{+} populations, Related to Figure 4.

(A) Experimental setup, see Figure 4A.
(B) Representative plots showing gating strategy for fraction of c-Myc^{+} cells among NP-Eα^{+} and NP-Eα^{-} LZ B1-8^{hi} cells.
(C) Representative flow cytometry histograms showing NP-Eα binding by c-Myc^{+} NP-Eα^{+} and c-Myc^{-} NP-Eα^{+} populations (left) and summary of gMFI intensity (right), two-tailed paired t-test, ****$p<0.0001$. Data from four independent experiments. Each dot represents one mouse. Lines depict mean.
(D) Experimental setup, see Figure 4C.
(E) Principal components analysis visualization of sorted populations.
(F) Gene set enrichment analysis (GSEA) summary of canonical pathways enriched in c-Myc^{+} NP-Eα^{+} compared to c-Myc^{+} NP-Eα^{-} populations, including Myc, mTOR, and NF-κB associated pathways.
(G) Expanded GSEA summary of hallmark pathways enriched in c-Myc^{+} NP-Eα^{+} compared to c-Myc^{+} NP-Eα^{-} populations, including Myc, cell cycle, and energy metabolism pathways.
(H) Expression of *Ccnd2* and *Batf* mRNA, one-way ANOVA with Tukey's multiple comparisons test, **$p=0.0014$, ****$p<0.0001$.
(I) Heatmap depicting expression of genes negatively correlated with c-Myc activity among c-Myc^{+} NP-Eα^{+} and c-Myc^{+} NP-Eα^{-} populations.
(J) Heatmap depicting expression of genes associated with pre- and memory B cell phenotype among c-Myc^{+} NP-Eα^{+} and c-Myc^{+} NP-Eα^{-} populations.
All enriched pathways had nominal p values<0.05 and FDR q values<0.25 (F and G). Each dot (E and H) and square (I and J) represents a sorted population of 400 cells. Each dot represents one Fab (E, F, and K). Lines depicts geometric mean (K).

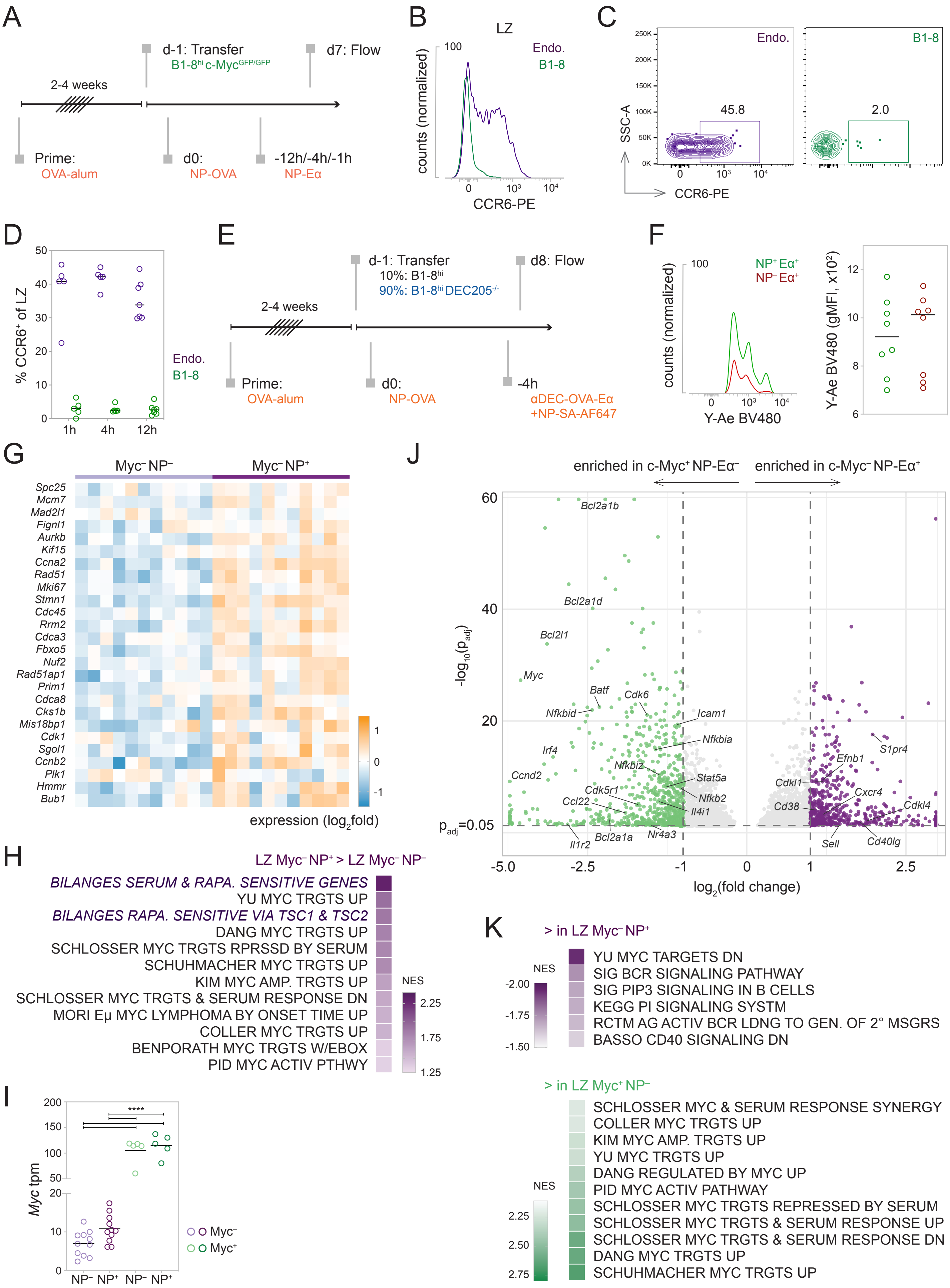

A
d-1: Transfer
B1-8hi c-MycGFP/GFP
d7: Flow
2-4 weeks
Prime:
OVA-alum
d0:
NP-OVA
-12h/-4h/-1h
NP-Eα
B
LZ
Endo.
B1-8
counts (normalized)
CCR6-PE
C
Endo.
B1-8
45.8
2.0
SSC-A
CCR6-PE
D
% CCR6+ of LZ
Endo.
B1-8
1h
4h
12h
E
d-1: Transfer
10%: B1-8hi
90%: B1-8hi DEC205-/-
d8: Flow
2-4 weeks
Prime:
OVA-alum
d0:
NP-OVA
-4h
αDEC-OVA-Eα
+NP-SA-AF647
F
NP+ Eα+
NP− Eα+
counts (normalized)
Y-Ae BV480
Y-Ae BV480 (gMFI, x10²)
G
Myc− NP−
Myc− NP+
Spc25
Mcm7
Mad2l1
Fignl1
Aurkb
Kif15
Ccna2
Rad51
Mki67
Stmn1
Cdc45
Rrm2
Cdca3
Fbxo5
Nuf2
Rad51ap1
Prim1
Cdca8
Cks1b
Mis18bp1
Cdk1
Sgol1
Ccnb2
Plk1
Hmmr
Bub1
expression (log2fold)
H
LZ Myc− NP+ > LZ Myc− NP−
BILANGES SERUM & RAPA. SENSITIVE GENES
YU MYC TRGTS UP
BILANGES RAPA. SENSITIVE VIA TSC1 & TSC2
DANG MYC TRGTS UP
SCHLOSSER MYC TRGTS RPRSSD BY SERUM
SCHUHMACHER MYC TRGTS UP
KIM MYC AMP. TRGTS UP
SCHLOSSER MYC TRGTS & SERUM RESPONSE DN
MORI Eμ MYC LYMPHOMA BY ONSET TIME UP
COLLER MYC TRGTS UP
BENPORATH MYC TRGTS W/EBOX
PID MYC ACTIV PTHWY
NES
I
Myc tpm
Myc−
Myc+
NP− NP+ NP− NP+
J
enriched in c-Myc+ NP-Eα−
enriched in c-Myc− NP-Eα+
-log10(padj)
padj=0.05
log2(fold change)
Bcl2a1b
Bcl2a1d
Bcl2l1
Myc
Batf
Cdk6
Nfkbid
Icam1
Nfkbia
Irf4
Nfkbiz
Stat5a
Ccnd2
Cdk5r1
Ccl22
Nfkb2
Il4i1
Il1r2
Bcl2a1a
Nr4a3
Cdkl1
Cd38
Sell
Efnb1
S1pr4
Cxcr4
Cdkl4
Cd40lg
K
> in LZ Myc− NP+
NES
YU MYC TARGETS DN
SIG BCR SIGNALING PATHWAY
SIG PIP3 SIGNALING IN B CELLS
KEGG PI SIGNALING SYSTM
RCTM AG ACTIV BCR LDNG TO GEN. OF 2° MSGRS
BASSO CD40 SIGNALING DN
> in LZ Myc+ NP−
NES
SCHLOSSER MYC & SERUM RESPONSE SYNERGY
COLLER MYC TRGTS UP
KIM MYC AMP. TRGTS UP
YU MYC TRGTS UP
DANG REGULATED BY MYC UP
PID MYC ACTIV PATHWAY
SCHLOSSER MYC TRGTS REPRESSED BY SERUM
SCHLOSSER MYC TRGTS & SERUM RESPONSE UP
SCHLOSSER MYC TRGTS & SERUM RESPONSE DN
DANG MYC TRGTS UP
SCHUHMACHER MYC TRGTS UP

Figure S4. Characterization of CCR6 expression, peptide presentation among LZ populations, and gene expression analysis of c-Myc⁻ populations, Related to Figure 4.

(A) Experimental setup. B1-8^{hi} c-Myc-GFP cells were transferred into OVA-primed mice and boosted with NP-OVA. NP-Eα was injected 12 h, 4 h, or 1 h before sacrifice.
(B-C) Representative histogram and (C) contour plots displaying CCR6 expression among endogenous or B1-8^{hi} LZ B cells.
(D) Frequencies of $CCR6^{+}$ cells among endogenous or B1-8^{hi} LZ B cells.
(E) Experimental setup for αDEC-OVA-Eα targeting. B1-8^{hi} and B1-8^{hi} $DEC205^{-/-}$ cells were transferred at a 10:90 ratio into OVA-primed hosts that were subsequently boosted with NP-OVA. 5 µg of αDEC-OVA-Eα was injected with NP-SA-AF647 4 h before sacrifice.
(F) Representative histogram (left) and summary (right) of Eα peptide presentation among LZ-NP-$E\alpha^{+}$ and -NP-$E\alpha^{-}$ populations following targeting, two-tailed paired t-test, ns.
(G) Heatmap depicting expression of genes associated with BCR stimulation among c-Myc^{-} NP-$E\alpha^{+}$ and c-Myc^{-} NP-$E\alpha^{-}$ populations.
(H) GSEA summary of canonical pathways enriched among c-Myc^{-} NP-$E\alpha^{+}$ compared to c-Myc^{-} NP-$E\alpha^{-}$ populations.
(I) Expression of *Myc* mRNA, one-way ANOVA with Tukey's multiple comparisons test, ****p<0.0001.
(J) Volcano plot depicting snapshot of differentially expressed genes between c-Myc^{+} NP-$E\alpha^{-}$ (green, left) and c-Myc^{-} NP-$E\alpha^{+}$ (purple, right) populations, genes with $p_{adj}>0.05$ not shown. Genes with $\log_2$(fold change) >3 and <-5 plotted as $\log_2(3)$ and $\log_2(-5)$, respectively. Genes with $-\log_{10}(p_{adj})>60$ plotted as $-\log_{10}(60)$.
(K) GSEA summary of canonical pathways enriched among c-Myc^{+} NP-$E\alpha^{-}$ compared to c-Myc^{-} NP-$E\alpha^{+}$ populations. Pathways depicted in purple (top) are enriched among c-Myc^{-} NP-$E\alpha^{+}$ cells and pathways depicted in green (bottom) are enriched in c-Myc^{+} NP-$E\alpha^{-}$ cells.
(H and K) All enriched pathways had nominal p values<0.05 and FDR q values<0.25. Lines depict means (D, F, and I). Data from two (D) and three (F) independent experiment, and each dot represents one mouse. Each square (G) and dot (I) represents a population of 400 cells.

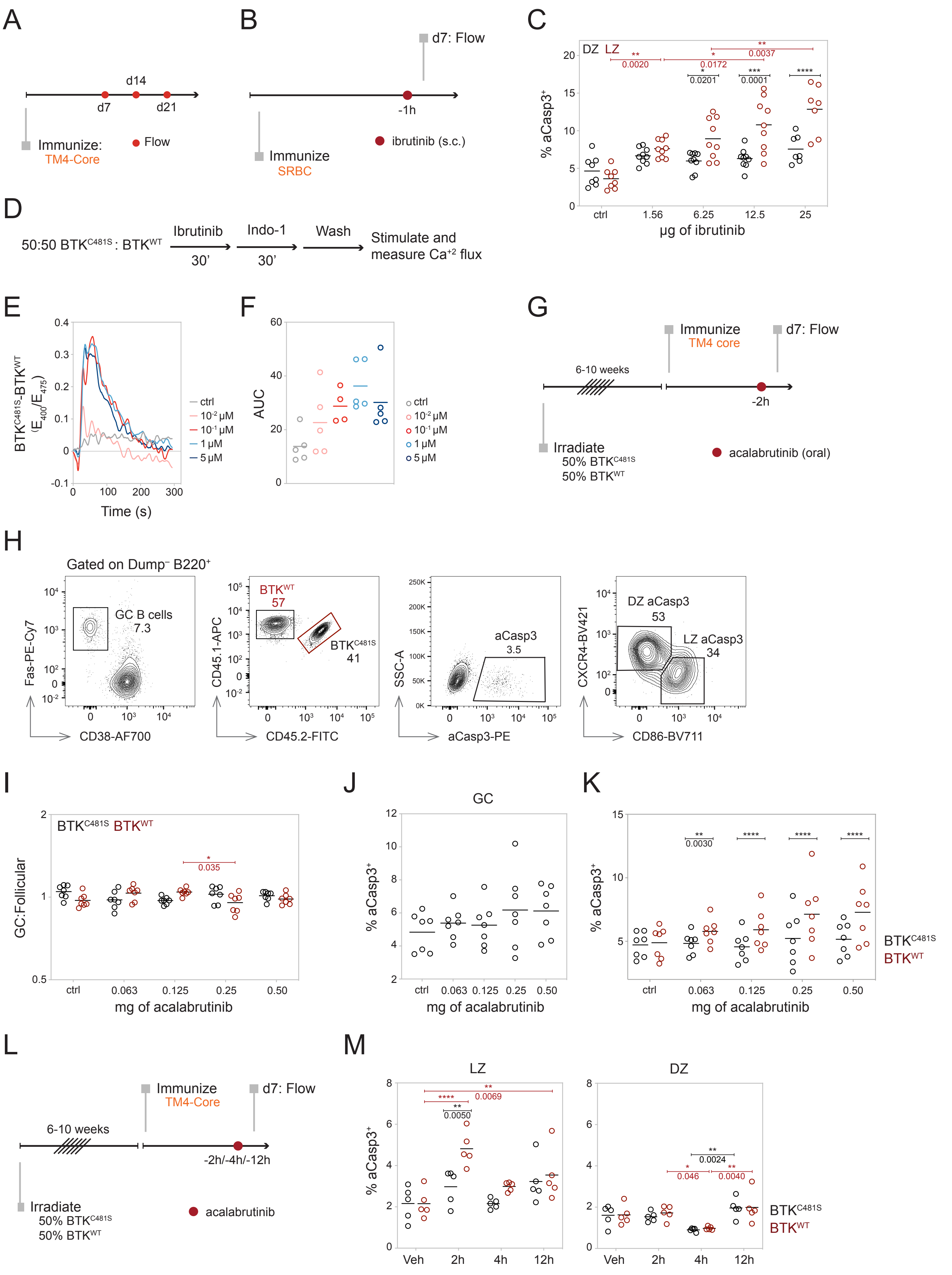
A
d14
d7
d21
Immunize:
TM4-Core
Flow
B
d7: Flow
-1h
Immunize
SRBC
ibrutinib (s.c.)
C
DZ LZ
% aCasp3+
0.0020
0.0172
0.0037
0.0201
0.0001
ctrl
1.56
6.25
12.5
25
μg of ibrutinib
D
50:50 BTK C481S : BTK WT
Ibrutinib
30'
Indo-1
30'
Wash
Stimulate and
measure Ca+2 flux
E
BTK C481S -BTK WT
(E400/E475)
Time (s)
ctrl
10-2 μM
10-1 μM
1 μM
5 μM
F
AUC
G
6-10 weeks
Immunize
TM4 core
d7: Flow
-2h
Irradiate
50% BTK C481S
50% BTK WT
acalabrutinib (oral)
H
Gated on Dump- B220+
GC B cells
7.3
Fas-PE-Cy7
CD38-AF700
BTK WT
57
BTK C481S
41
CD45.1-APC
CD45.2-FITC
aCasp3
3.5
SSC-A
aCasp3-PE
DZ aCasp3
53
LZ aCasp3
34
CXCR4-BV421
CD86-BV711
I
BTK C481S BTK WT
0.035
GC:Follicular
ctrl
0.063
0.125
0.25
0.50
mg of acalabrutinib
J
GC
% aCasp3+
mg of acalabrutinib
K
% aCasp3+
0.0030
mg of acalabrutinib
BTK C481S
BTK WT
L
6-10 weeks
Immunize
TM4-Core
d7: Flow
-2h/-4h/-12h
Irradiate
50% BTK C481S
50% BTK WT
acalabrutinib
M
LZ
DZ
% aCasp3+
0.0069
0.0050
0.0024
0.046
0.0040
Veh
2h
4h
12h
BTK C481S
BTK WT

Figure S5. Characterization of BTK^{C481S} mutant and BTK inhibition differentially affects LZ cells, Related to Figure 5.

(A) TM4-Core immunization, see Figures 5D and 5E.
(B) Sheep red blood cell (SRBC) immunization with ibrutinib s.c. pulse.
(C) Frequency of $aCasp3^{+}$ cells among LZ and DZ populations one hour after ibrutinib treatment, two-way ANOVA with Šidák's multiple comparisons (within dose) and Tukey's multiple comparisons (across doses), *, **, and ***p values as marked, ****p<0.0001.
(D) Schematic depicting setup for ex vivo Ca++ flux assay.
(E) Ca++ flux at increasing concentrations of ibrutinib treatment, depicted as subtraction of BTK^{WT} E_{400}/E_{450} trace from BTK^{C481S} E_{400}/E_{450} trace (BTK^{C481S}– BTK^{WT}).
(F) AUC of individual BTK^{C481S}– BTK^{WT} traces.
(G) TM4-core immunization of mixed BTK^{C481S}:BTK^{WT} bone marrow (BM) chimeras with different doses of acalabrutinib.
(H) Gating strategy for mixed BM chimera $aCasp3^{+}$ analysis shown in (Figure 5G).
(I) GC to follicular B cell ratios of BTK^{C481S}:BTK^{WT} mixed BM chimeras, two-way ANOVA with Tukey's multiple comparisons test, *p=0.035.
(J) $aCasp3^{+}$ frequency of total GC, two-way ANOVA with Šidák's multiple comparisons, ns.
(K) $aCasp3^{+}$ frequency of GC BTK^{C481S} and GC BTK^{WT} cells with acalabrutinib treatment, RM two-way ANOVA with Šidák's multiple comparisons, **p=0.0030, ****p<0.0001 (within dose), Tukey's multiple comparisons (across doses), ns.
(L) Experimental setup for TM4-core immunization of mixed BTK^{C481S}:BTK^{WT} BM chimeras with 0.0625 mg acalabrutinib treatment (oral) for different timepoints.
(M) Frequency of $aCasp3^{+}$ cells among LZ and DZ BTK^{C481S} and BTK^{WT} cells across timepoints, two-way ANOVA with Šidák's multiple comparisons (within timepoint) or Tukey's multiple comparisons (across timepoints), * and **p values as marked, ****p<0.0001.
(C, I, J, and L) Data from two independent experiments, each dot represents one mouse. Data from two independent experiments, each dot represents one sample (F). (C, F, I, J, and L) Lines depict means.

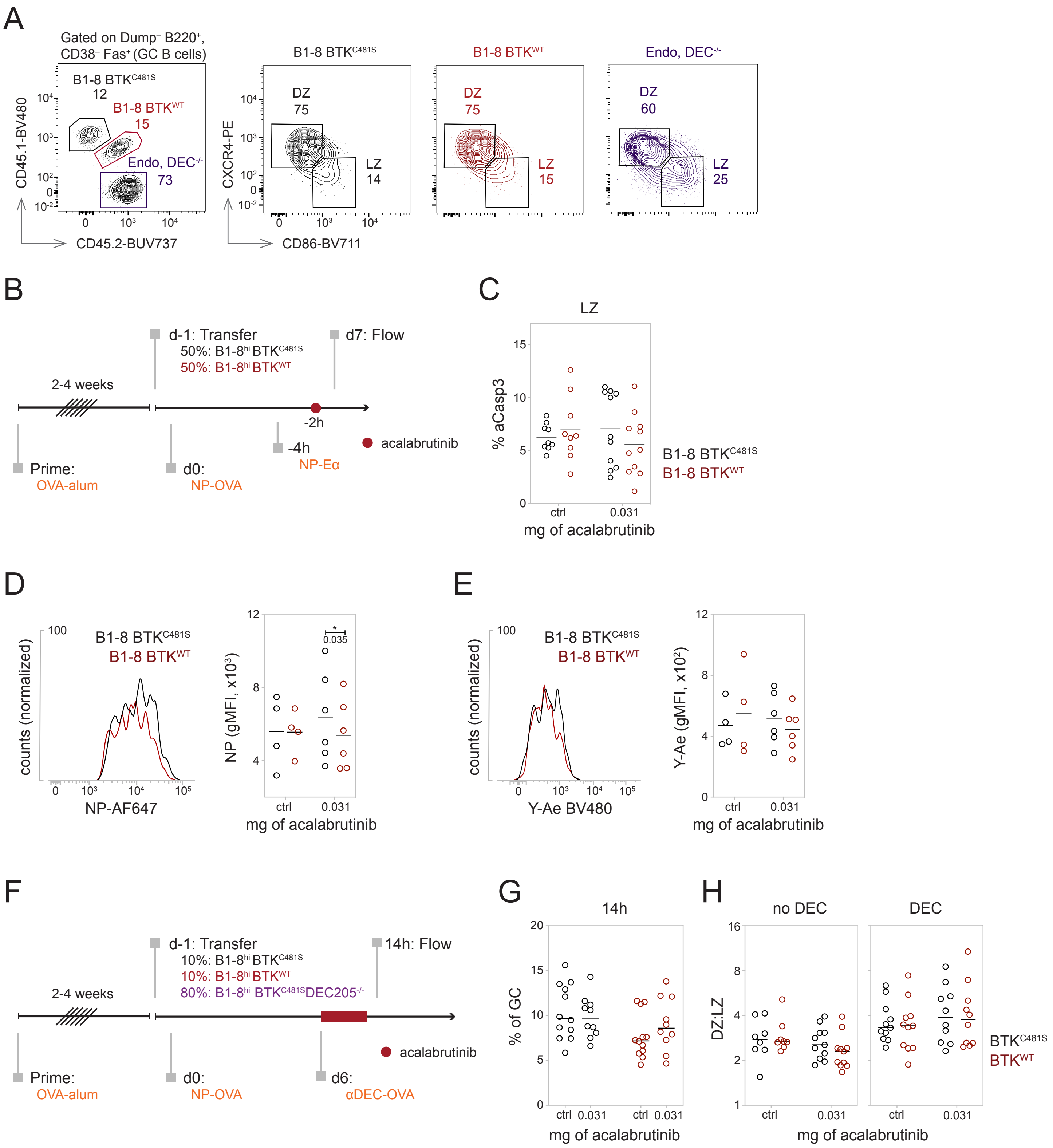
A
Gated on Dump⁻ B220⁺, CD38⁻ Fas⁺ (GC B cells)
B1-8 BTKC481S 12
B1-8 BTKWT 15
Endo, DEC-/- 73
CD45.1-BV480
CD45.2-BUV737
B1-8 BTKC481S
DZ 75
LZ 14
B1-8 BTKWT
DZ 75
LZ 15
Endo, DEC-/-
DZ 60
LZ 25
CXCR4-PE
CD86-BV711
B
d-1: Transfer
50%: B1-8hi BTKC481S
50%: B1-8hi BTKWT
d7: Flow
2-4 weeks
-2h
-4h
NP-Eα
acalabrutinib
Prime:
OVA-alum
d0:
NP-OVA
C
LZ
% aCasp3
ctrl
0.031
mg of acalabrutinib
B1-8 BTKC481S
B1-8 BTKWT
D
counts (normalized)
B1-8 BTKC481S
B1-8 BTKWT
NP-AF647
NP (gMFI, x10³)
*
0.035
ctrl
0.031
mg of acalabrutinib
E
counts (normalized)
B1-8 BTKC481S
B1-8 BTKWT
Y-Ae BV480
Y-Ae (gMFI, x10²)
ctrl
0.031
mg of acalabrutinib
F
d-1: Transfer
10%: B1-8hi BTKC481S
10%: B1-8hi BTKWT
80%: B1-8hi BTKC481SDEC205-/-
14h: Flow
2-4 weeks
acalabrutinib
Prime:
OVA-alum
d0:
NP-OVA
d6:
αDEC-OVA
G
14h
% of GC
ctrl
0.031
ctrl
0.031
mg of acalabrutinib
H
no DEC
DEC
DZ:LZ
ctrl
0.031
ctrl
0.031
mg of acalabrutinib
BTKC481S
BTKWT

Figure S6. Treatment with low doses of acalabrutinib dampens BCR signaling without affecting LZ cell survival, Related to Figure 6.

(A) Example gating strategy, see Figures 6A and 6B.
(B) Experimental setup for prime-boost and mixed transfer of B1-8^{hi} BTK^{C481S} and B1-8^{hi} BTK^{WT} cells. Four hours prior to readout, NP-Eα was injected and followed by oral gavage with acalabrutinib (0.03125 mg) two hours later.
(C) Frequency of LZ aCasp3^{+} cells among B1-8^{hi} BTK^{C481S} and B1-8^{hi} BTK^{WT} populations, RM two-way ANOVA with Šidák's multiple comparisons, ns.
(D-E) Representative histogram (left) and summary of gMFIs (right) for NP-AF647 and (E) Y-Ae-BV480, comparing LZ B1-8^{hi} BTK^{C481S} and B1-8^{hi} BTK^{WT} populations, RM two-way ANOVA with Šidák's multiple comparisons, (D) *p=0.035 and (E) ns.
(F) Experimental setup for prime-boost and mixed transfer of B1-8^{hi} BTK^{C481S}, B1-8^{hi} BTK^{WT}, and B1-8^{hi} BTK^{C481S} DEC205-/- cells. αDEC-OVA (t=0 h) was injected followed by treatment with 3 doses of 0.03125 mg of acalabrutinib, or vehicle, by oral gavage (t=0, 6, and 12 h). Mice were sacrificed two hours after final acalabrutinib treatment (t=14 h).
(G) Frequencies of B1-8^{hi} BTK^{C481S} and B1-8^{hi} BTK^{WT} populations treated as in (F).
(H) DZ:LZ ratios of B1-8^{hi} BTK^{C481S} and B1-8^{hi} BTK^{WT} populations 60 hours after treatment with PBS (no DEC, left) or αDEC-OVA (DEC, right) from experiment as shown in Figure 6A. Mice were treated with vehicle or 0.03125 mg of acalabrutinib as indicated in Figure 6A, RM two-way ANOVA with Šidák's multiple comparisons (G and H), ns.
Data from four (C, G, and H) and two (D and E) independent experiments. (C, D, E, G, and H) Each dot represents one mouse and lines represent means.

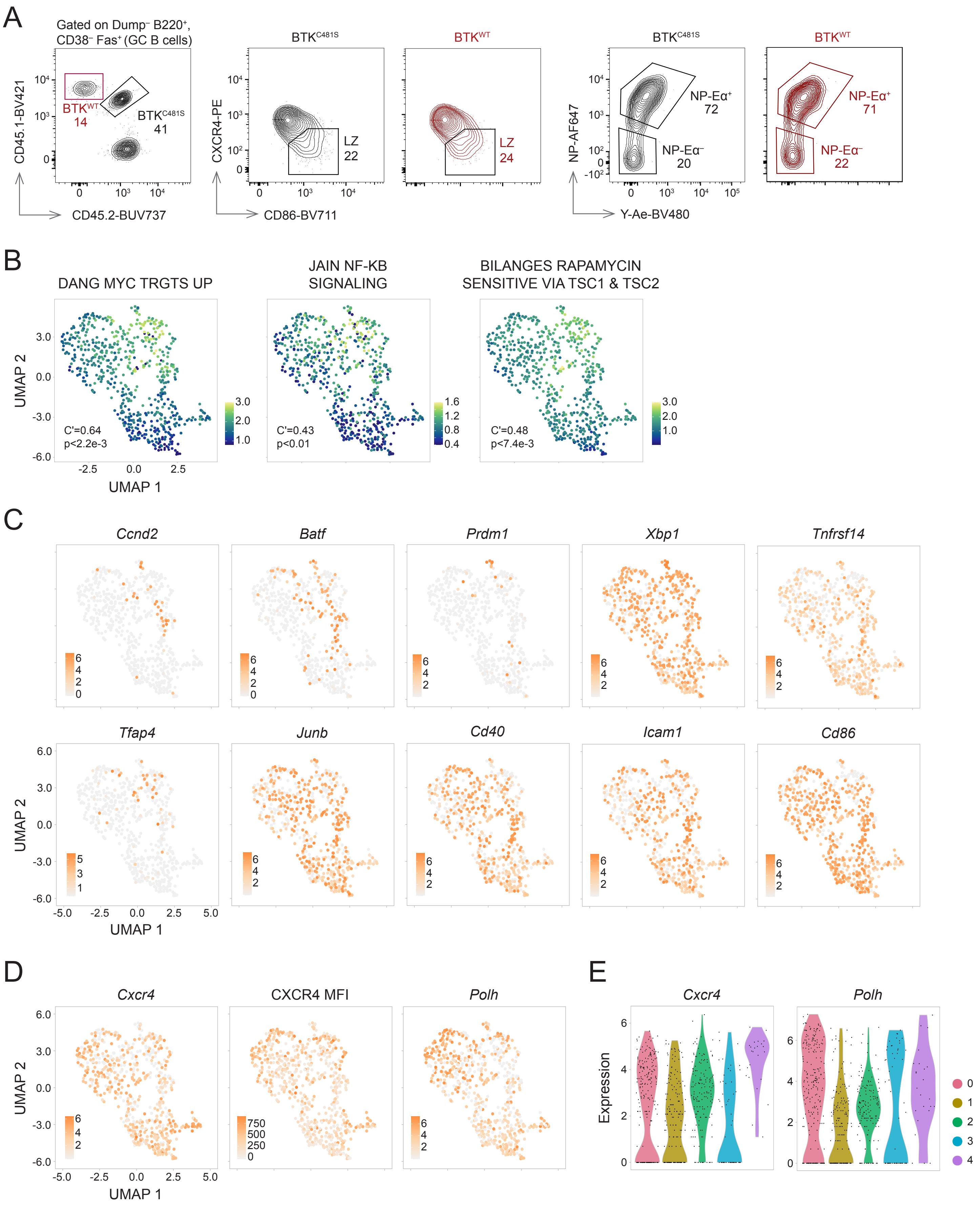
A
Gated on Dump⁻ B220⁺, CD38⁻ Fas⁺ (GC B cells)
CD45.1-BV421
CD45.2-BUV737
BTKWT 14
BTKC481S 41
BTKC481S
BTKWT
CXCR4-PE
CD86-BV711
LZ 22
LZ 24
NP-AF647
Y-Ae-BV480
NP-Eα⁺ 72
NP-Eα⁻ 20
NP-Eα⁺ 71
NP-Eα⁻ 22
B
DANG MYC TRGTS UP
JAIN NF-KB SIGNALING
BILANGES RAPAMYCIN SENSITIVE VIA TSC1 & TSC2
UMAP 2
UMAP 1
C'=0.64 p<2.2e-3
C'=0.43 p<0.01
C'=0.48 p<7.4e-3
C
Ccnd2
Batf
Prdm1
Xbp1
Tnfrsf14
Tfap4
Junb
Cd40
Icam1
Cd86
D
Cxcr4
CXCR4 MFI
Polh
E
Cxcr4
Polh
Expression
0
1
2
3
4

Figure S7. BTK inhibition sorting for single-cell RNA-seq and functional interpretations of clusters, Related to Figure 6.

(A) Sorting Strategy. LZ-B1-8^{hi} BTK^{C481S} and -B1-8^{hi} BTK^{WT} B cells were sorted from experiment as shown in Figure 6C.
(B) Enrichment of c-Myc targets,[46] NF-κB signaling,[47] and mTOR pathways[48] visualized on UMAP by signature scores.
(C) Expression of genes associated with T cell positive selection: *Tfap4*, *Ccnd2*, *Batf*, and *Junb*; genes associated with plasma cell differentiation: *Prdm1* and *Xbp1*; and expression of co-stimulatory molecules: *Cd40*, *Icam1*, *Cd86*, and *Tnfrsf14*.
(D) Expression of markers associated with DZ phenotype: *Cxcr4* gene expression (left), CXCR4 surface staining (middle), and *Polh* gene expression (right).
(E) Violin plot depicting expression of *Cxcr4* and *Polh* across clusters.
Autocorrelation and p values depicted on graphs (B).
